# Supplementary material for: Deep whole-genome sequencing of 3 cancer cell lines on 2 sequencing platforms
Source: Sci Rep. 2019 Dec 13;9:19123. doi: 10.1038/s41598-019-55636-3 (PMC6911065; doi:10.1038/s41598-019-55636-3)
Supplement: Supplementary file 1 — Supplemental Information [file 41598_2019_55636_MOESM1_ESM.pdf]

Deep whole-genome sequencing of 3 cancer cell lines on 2 sequencing platforms

Kanika Arora[1], Minita Shah[1], Molly Johnson[1], Rashesh Sanghvi[1], Jennifer Shelton[1], Kshithija Nagulapalli[1], Dayna M. Oschwald[1], Michael C. Zody[1], Soren Germer[1], Vaidehi Jobanputra[1], Jade Carter[1], Nicolas Robine[1,*]

[1] New York Genome Center, New York, NY 10013, USA

Supplemental information

Supplemental File: Pipeline diagram and commands (HTML)
Supplemental Table 1: Cell line passage information from ATCC.
Supplemental Table 2: Alignment metrics and duplication rates.
Supplemental Figure S1: Karyotypes of COLO-829, HCC-1187, HCC-1143 and its associated “normal” cell lines HCC-1143BL.
Supplemental Figure S2: Base quality scores by cycle, before and after BQSR.
Supplemental Figure S3: Fraction of total reads containing homopolymer (stretches of 20nt or longer).
Supplemental Figure S4: Intra-run and inter-platform concordance of somatic variants called by the different variant callers, similar to figure 1.
Supplemental Figure S5: Mutation spectrum of concordant high confidence SNVs between HiSeqX and Novaseq.
Supplemental Figure S6: Single nucleotide mismatches by type in samples sequenced on NovaSeq and HiSeqX.
Supplemental Figure S7: Difference in the fraction of mismatches between HiSeqX and NovaSeq per trinucleotide.
Supplemental Figure S8: Difference in the mismatches between HiSeqX and NovaSeq per trinucleotide collapsed to the 6 mismatch categories(C>A, C>G, C>T, T>A, T>C, T>G).
Supplemental Figure S9: Allele frequency and mutational spectrum of discordant SNVs between HiSeqX and NovaSeq without Panel of Normal filtering.
Supplemental Figure S10: Allele frequency and mutational spectrum of discordant high confidence SNVs between HiSeqX and NovaSeq.
Supplemental Figure S11: Sources of discrepancies between NYGC callset and the reference dataset established in Craig et al.
Supplemental Figure S12: Adjustment of Log2 Values in Cell Line Purity Ladder.
Supplemental Figure S13: Variant allele frequency distribution and number of high confidence SNVs and Indels called in the high coverage data that are also called in the AllSomatic callsets of the purity ladder samples for (A) COLO-829 and (B) HCC-1143.
Supplemental Figure S14: Precision, recall and F1 scores at different simulated purities for CNVs without (Original) and with (CELLULOID/HATCHet) adjustments of log2 values for purity and ploidy.
Supplemental Figure S15 Precision, recall and F1 scores for AllSomatic and HighConfidence SNV, INDEL and SV callsets at different coverages of tumor and normal data from COLO-829 (top) and HCC-1143 (bottom).
Supplemental Figure S16: (A) Recall of SNVs and Indels in different variant allele frequency ranges, for different tumor and normal coverages of COLO-829 (left) and HCC-1143 (right).
Supplemental Figure S17: Number of true positive variants called on the purity ladder samples in AllSomatic and HighConf callsets of the NYGC pipeline, and by individual callers.
Supplemental Figure S18:(A) Number of calls made when we treated 90X average coverage COLO-829BL (normal) cell line data as “tumor”, and a distinct set of reads from the same cell line at 40X average coverage as “normal”.(B) UpSet plots that show the number of variants removed from the AllSomatic callset by each filtering step.

Supplemental File: pipeline_specs.zip, containing 3 HMTL files (and 3 corresponding diagrams in the folder figs) describing the pipeline, the preprocessing steps and the calling steps in great details.

Cell line	Ampule passage number (from ATCC)
COLO-829	10
HCC-1143	5
HCC-1187	28

Supplemental Table1: Cell line passage information from ATCC

Sample	Platform	Total Reads	%Aligned Reads	Mean Coverage	%Duplicates
COLO-829	HiSeqX	3942506750	99.64	166.36	11.62
COLO-829	NovaSeq	5111570566	99.57	228.06	6.28
COLO-829BL	HiSeqX	2125523908	99.64	89.93	11.18
COLO-829BL	NovaSeq	4062312284	99.62	179.73	6.97
HCC-1143	HiSeqX	1928441034	99.61	81.15	11.65
HCC-1143	NovaSeq	6310318566	99.54	278.26	7.28
HCC-1143BL	HiSeqX	1017638416	99.53	42.35	11.84
HCC-1143BL	NovaSeq	3566322944	99.63	155.73	7.47
HCC-1187	HiSeqX	1914759882	99.69	79.80	11.35
HCC-1187	NovaSeq	2056483546	99.71	90.35	6.43
HCC-1187BL	HiSeqX	1016297632	99.63	42.58	11.24
HCC-1187BL	NovaSeq	1390489154	99.65	61.47	6.22

Supplemental Table2: Alignment metrics and duplication rates

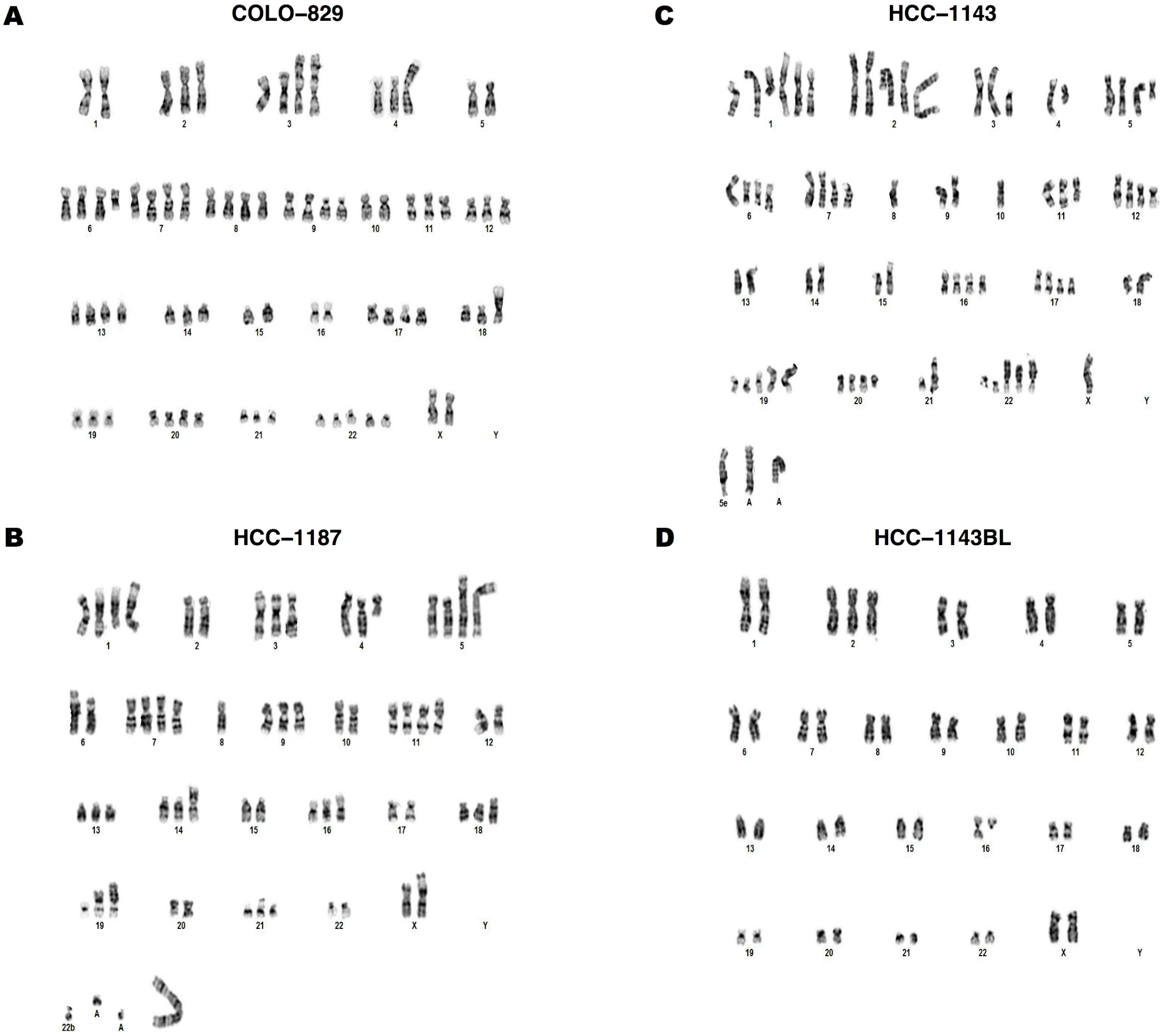

Supplemental Figure S1: Karyotypes of COLO-829, HCC-1187, HCC-1143 and its associated “normal” cell lines HCC-1143BL. We note some slight differences between the results of the karyotype analyses and the CNV analyses resulting from WGS, possibly due to clonal heterogeneity, technical differences and differences in the level of detection of the technologies.

(A) COLO-829: 70~73<3N>,XX,-1,del(1)(q12),+3,der(3)t(1;3)(q12;p25)x2,i(4)(q10),-5,+6,del(6)(q13q25),+7,dup(7)(q32q34)x2,+8,+9,del(9)(p11.2)x2,-10,+13,-15,-16,+17,der(18)t(1;18)(p21;p11.3),+20,+22,+22,+22 [cp20]

(B) HCC-1187: 63~67<3N>,X,add(X)(p22.1),+add(1)(p22),+add(1)(p34),del(1)(q21),del(1)(q32),-2,del(2)(p13p23)x2,+3,del(3)(p13),i(5)(q10)x2,del(5)(q13q33),del(6)(q13),+7,-8,del(8)(q22),-10,+11,add(11)(p15),add(12)(q22),del(13)(q22q32)x3,add(16)(q24),del(17)(p11.2),add(18)(q23),+19,add(19)(p13)x2,-20,add(20)(q13.3),+21,+4~6mar [cp20]

(C) HCC-1143: 74~82<3N>,X,+add(1)(p34),+add(1)(q21),+del(1)(p32p34),+2,add(2)(q31),del(3)(p13),+4,del(4)(q22)x2,+5,del(5)(q13q33),add(7)(q22),del(7)(p13),-8,-10,+11,del(11)(q13q23),del(11)(q23q24),del(12)(q13q22),-14,add(14)(p11.2),del(17)(p11.2),+17,add(18)(p11.2),+19,add(19)(p13.3),add(21)(q22),+4~5mar [cp20]

(D) HCC-1143BL: 47,XX,+2 [15]/47,XX,+2,del(16)(q12) [5]

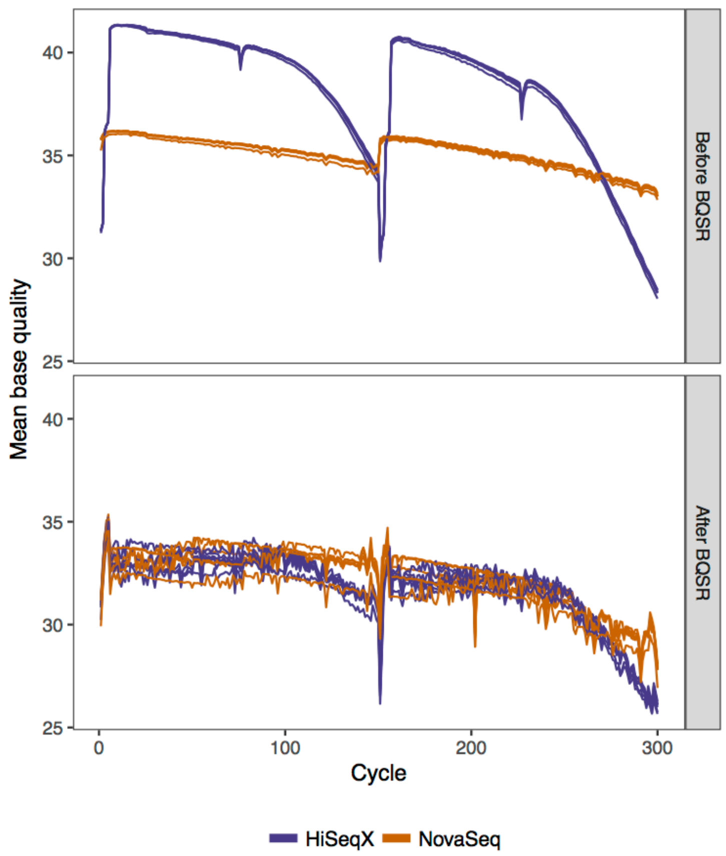

Supplemental Figure S2: Base quality scores by cycle, before and after BQSR.

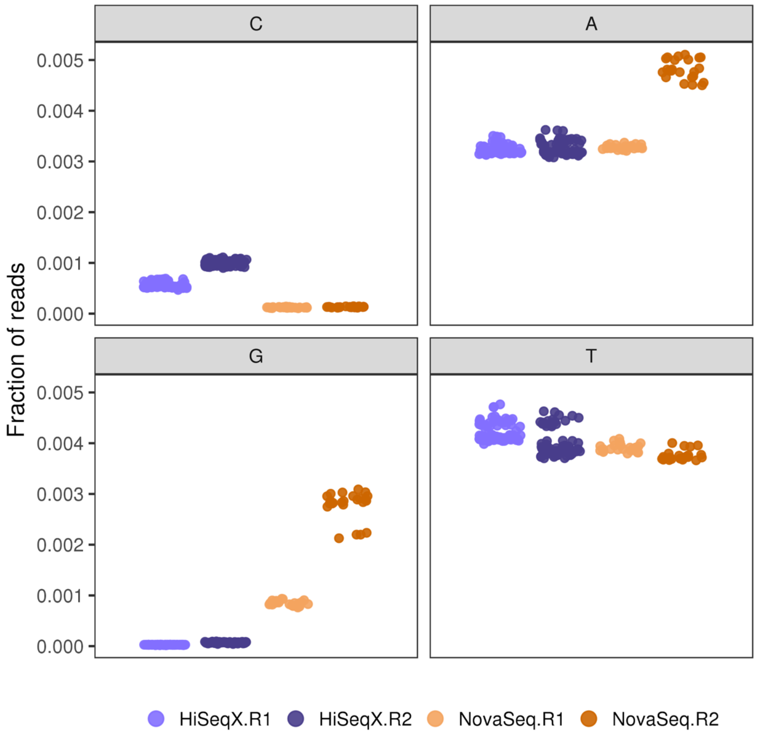

Supplemental Figure S3: Fraction of total reads containing homopolymer (stretches of 20nt or longer)

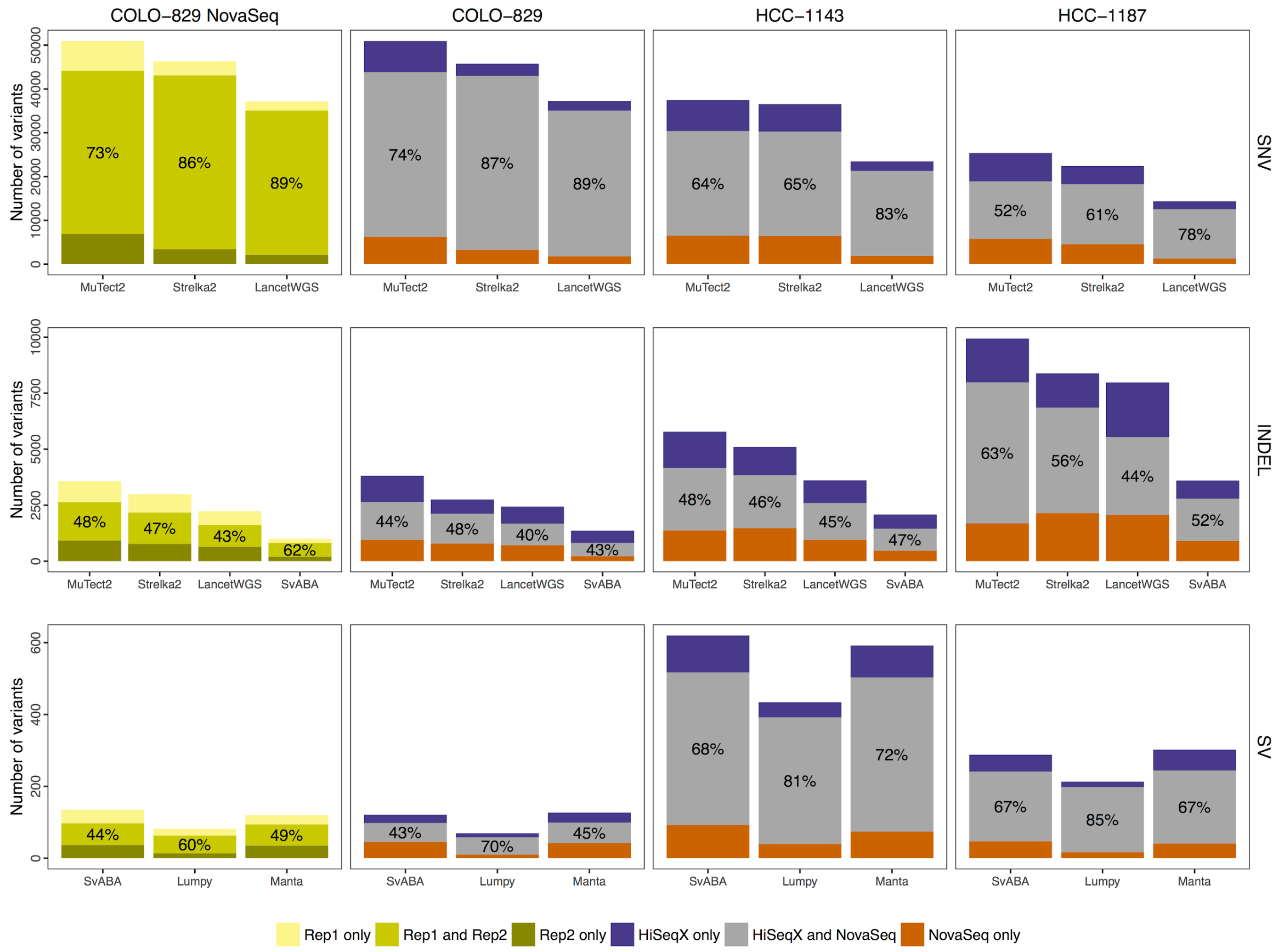

Supplemental Figure S4: Intra-run and inter-platform concordance of somatic variants called by the different variant callers, similar to figure 2. Even though Lancet is run in Lancet exonic and validation modes in the pipeline, for this plot, we show the results of Lancet run on the entire genome.

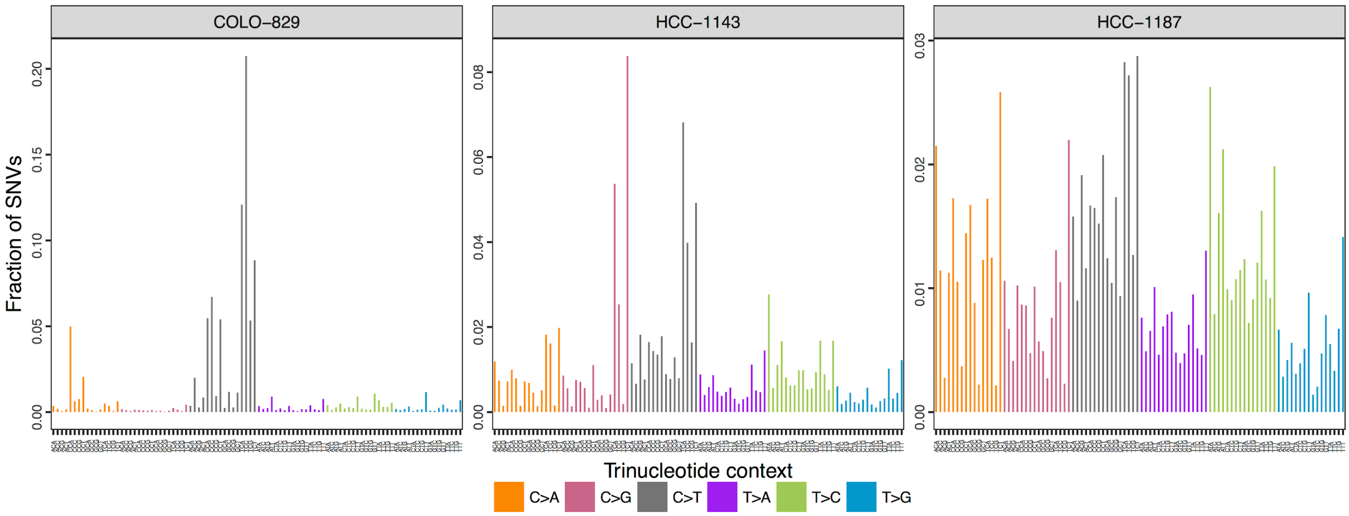

Supplemental Figure S5**:** Mutation spectrum of concordant high confidence SNVs between HiSeqX and Novaseq.

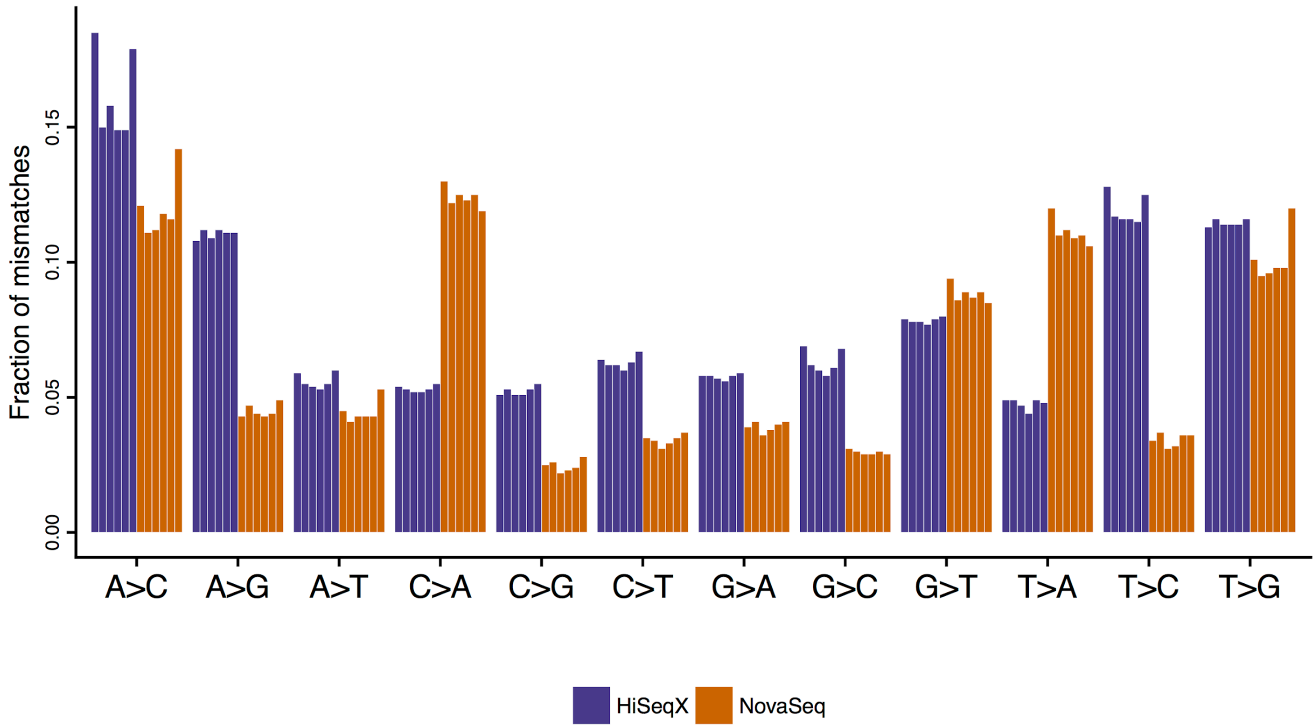

Supplemental Figure S6: Single nucleotide mismatches by type in samples sequenced on NovaSeq and HiSeqX. We find that NovaSeq had more C>A and T>A mismatches, whereas HiSeqX had more A>G and T>G mismatches. Each bar represents a single sample and colored based on sequencing platform. HiSeqX samples had an average mismatch rate of 0.75%, whereas NovaSeq samples had average mismatch rates of 0.6%.

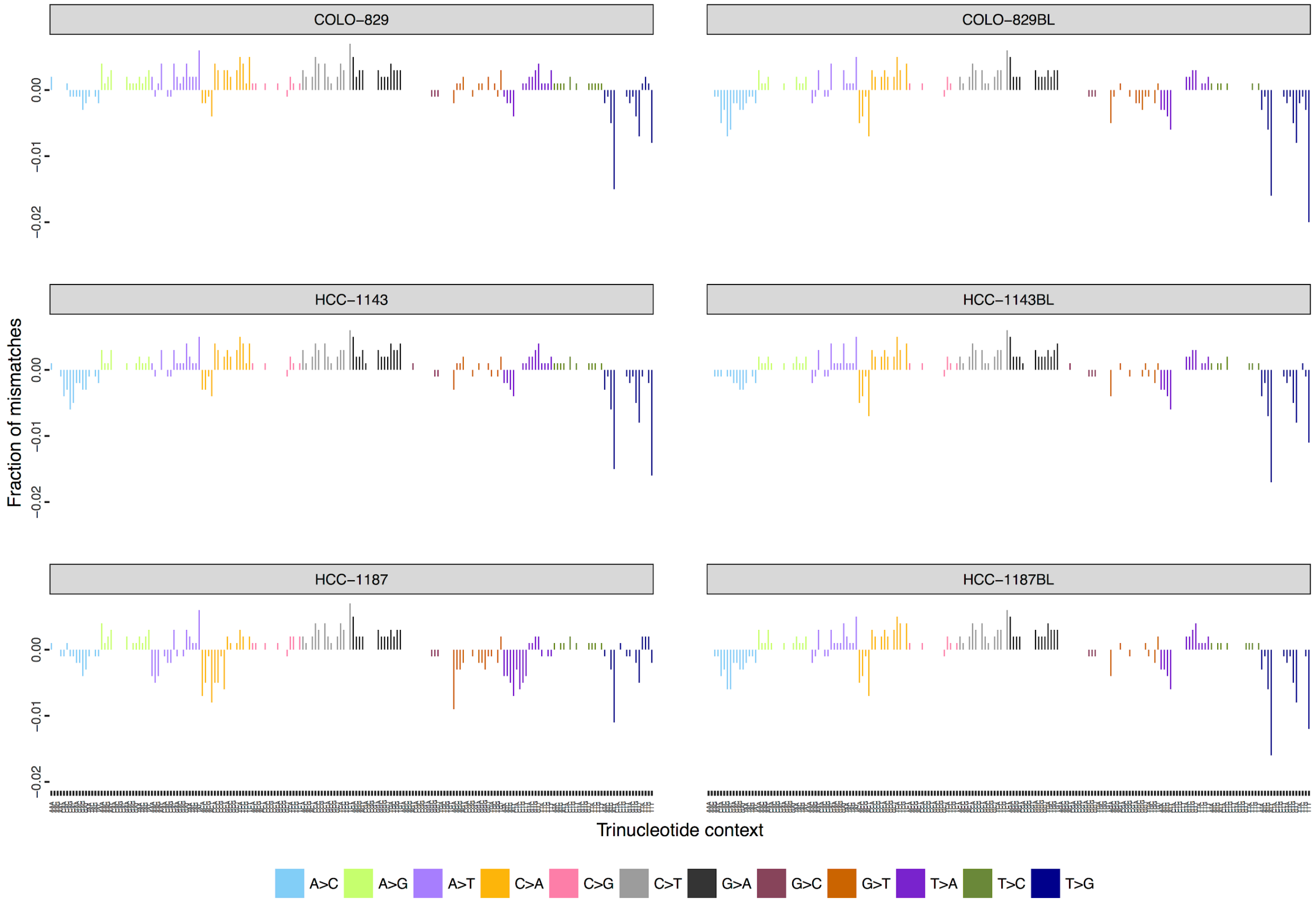

Supplemental Figure S7**:** Difference in the fraction of mismatches between HiSeqX and NovaSeq per trinucleotide. Positive values correspond to higher fractions in HiSeqX and negative values correspond to higher fractions in NovaSeq. MQ ≥ 10 and BQ≥10 cut-offs were applied for this calculation. We observed that NovaSeq called more T>G mismatches, especially in A [T>G]T, G [T>G]T and T [T>G]T context.

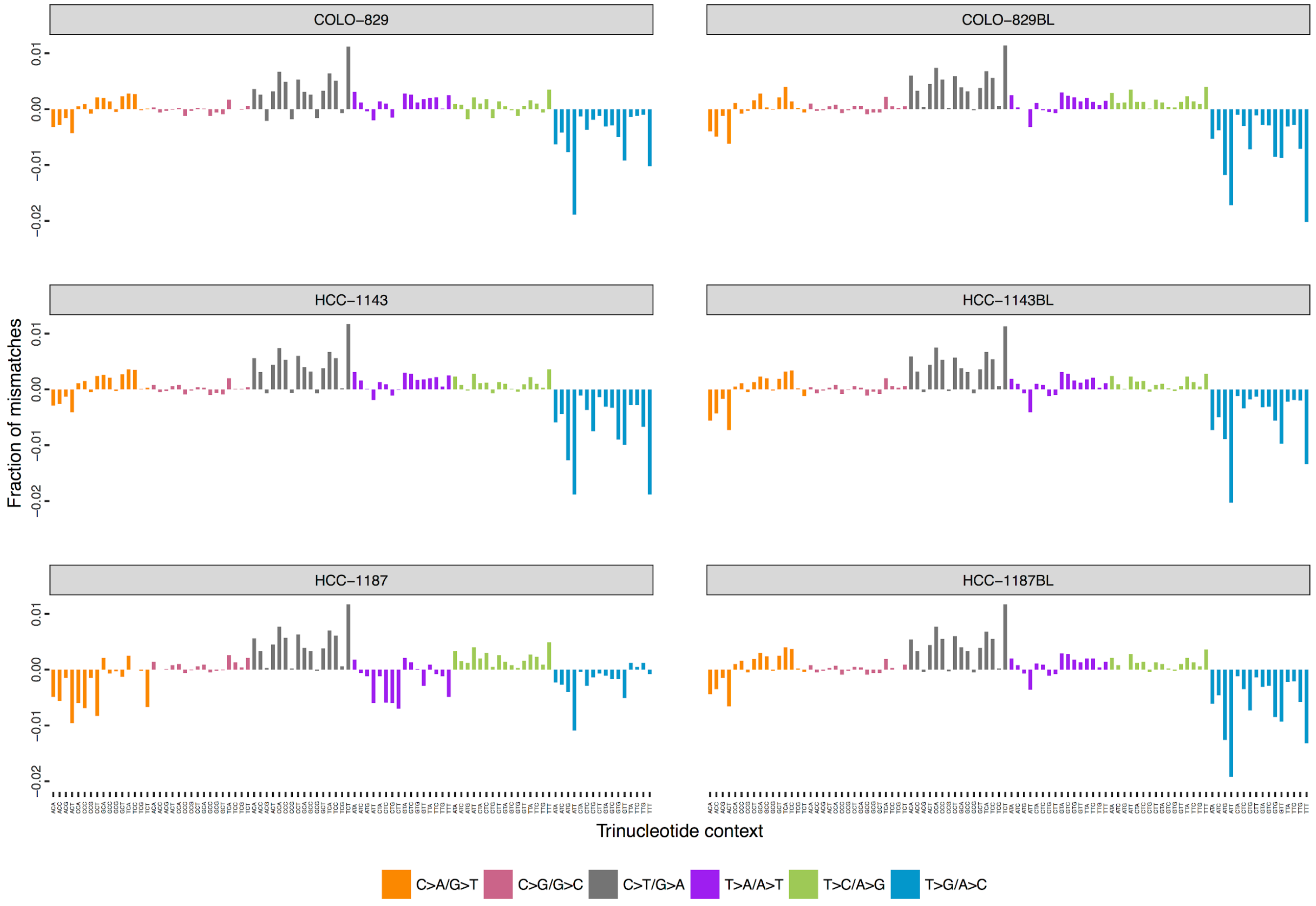

Supplemental Figure S8: Difference in the mismatches between HiSeqX and NovaSeq per trinucleotide collapsed to the 6 mismatch categories(C>A, C>G, C>T, T>A, T>C, T>G). Positive values correspond to higher fractions in HiSeqX and negative values correspond to higher fractions in NovaSeq. MQ ≥ 10 and BQ≥10 cut-offs were applied for this calculation. We observe that NovaSeq called more T>G mismatches, especially in A [T>G]T, G [T>G]T and T [T>G]T context.

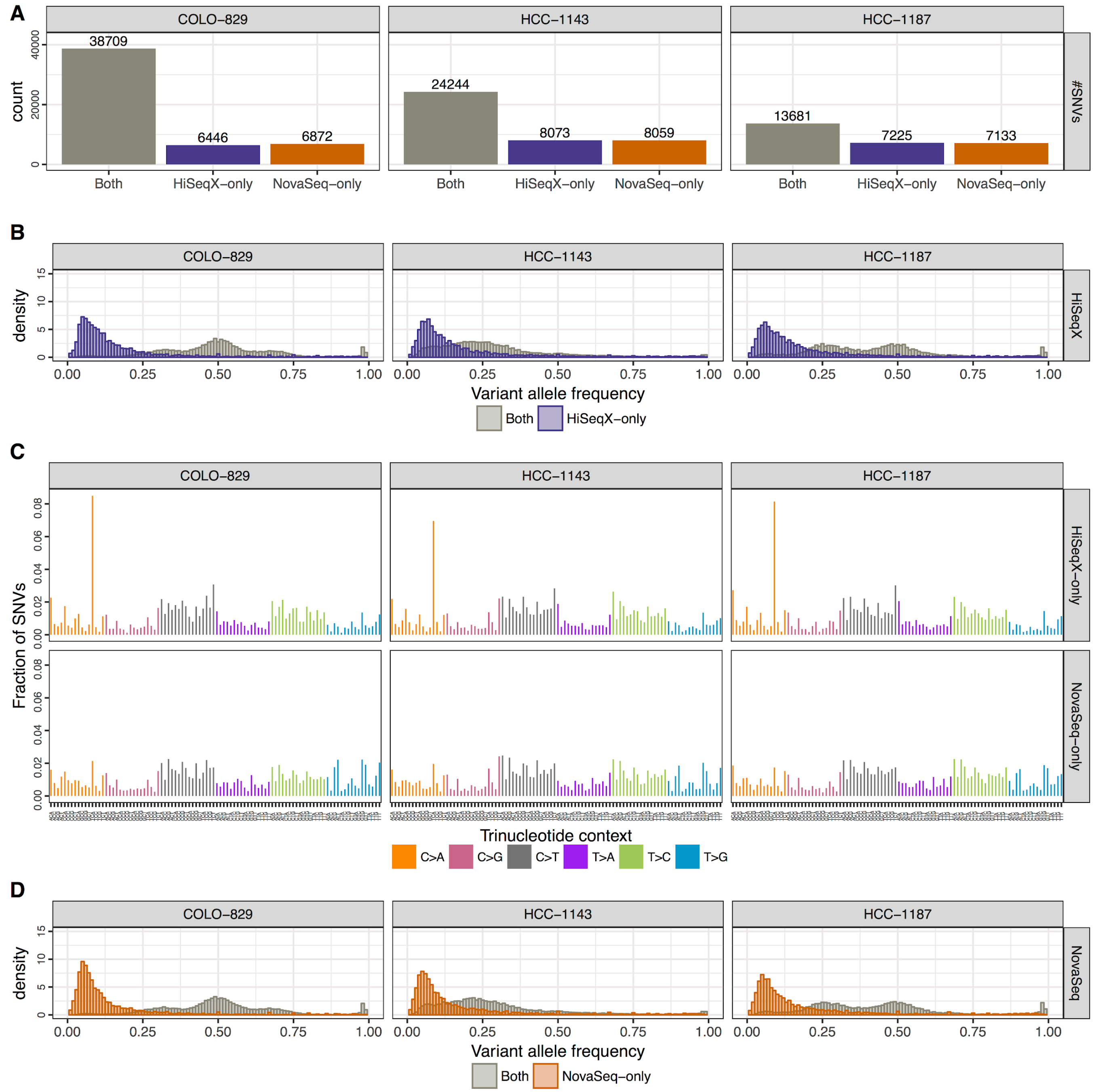

Supplemental Figure S9: Allele frequency and mutational spectrum of discordant SNVs between HiSeqX and NovaSeq without Panel of Normal filtering. Panel A shows the number of SNVs that were called in both NovaSeq and HiSeqX data, only in HiSeqX data and only in NovaSeq data. Panel B shows the allele frequency of the variants called only by HiSeqX in purple, and for reference the allele frequency of variants called by both platforms. Panel C shows the decomposition in trinucleotide contexts of the variants called uniquely by each platform (top and bottom tracks) and called by both platform (middle track). Panel D is similar to Panel B but for variants uniquely called by NovaSeq.

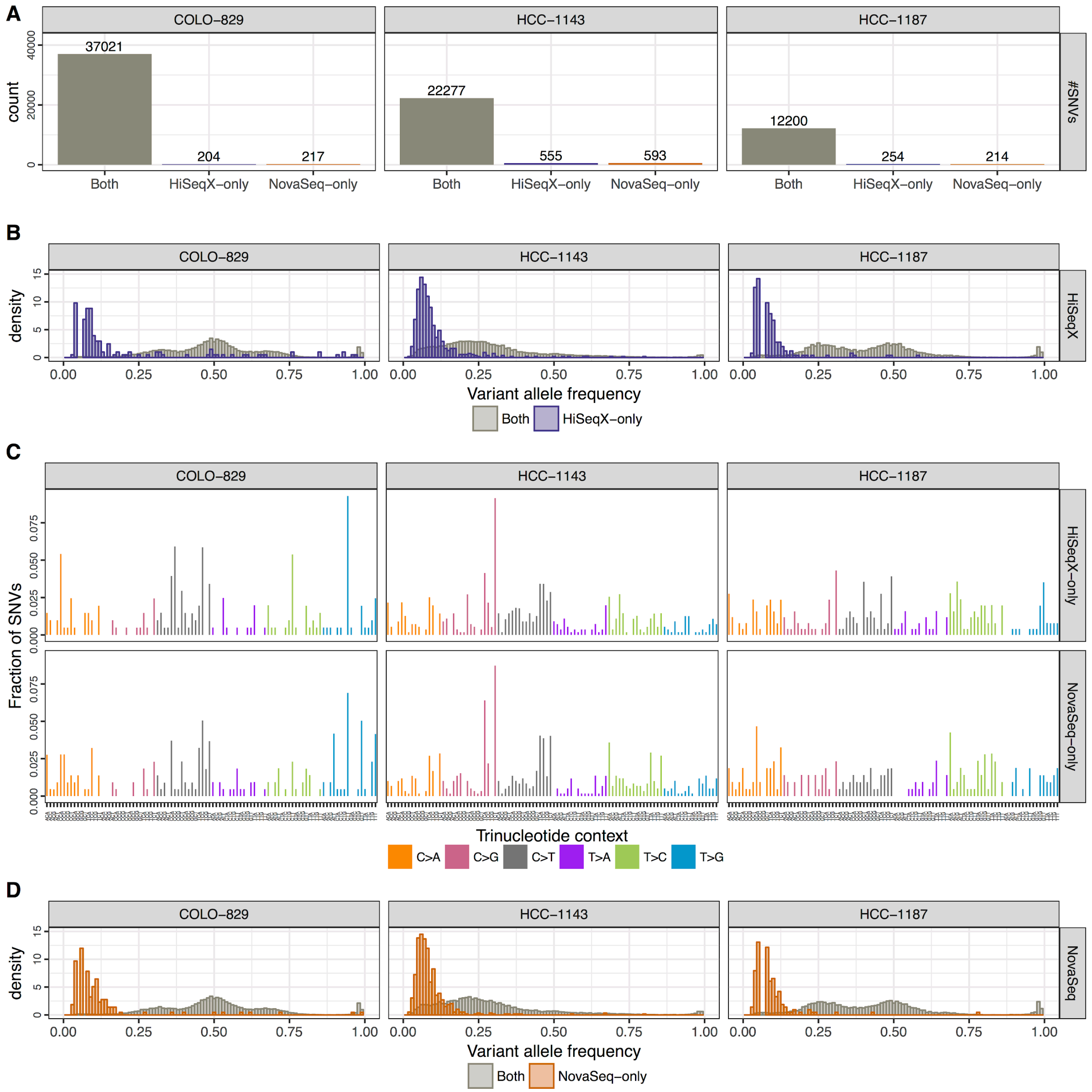

Supplemental Figure S10: Allele frequency and mutational spectrum of discordant high confidence SNVs between HiSeqX and NovaSeq. Only those SNVs that were in the high confidence callset for at least one of the technologies were used for this. Panel A shows the number of SNVs that were called in both NovaSeq and HiSeqX data, only in HiSeqX data and only in NovaSeq data. Panel B shows the allele frequency of the variants called only by HiSeqX in purple, and for reference the allele frequency of variants called by both platforms. Panel C shows the decomposition in trinucleotide contexts of the variants called uniquely by each platform. Panel D is similar to Panel B but for variants uniquely called by NovaSeq.

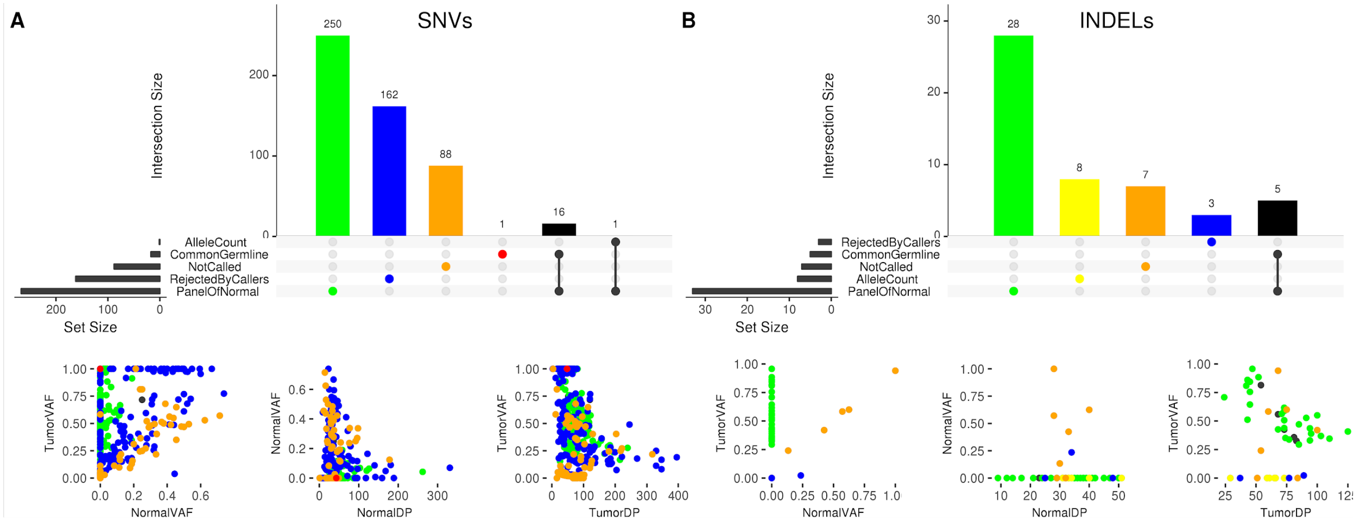

Supplemental Figure S11: Sources of discrepancies between NYGC callset and the reference dataset established in Craig et al. The figure shows (A) SNVs and (B) Indels from Craig et al. dataset that were not called in our AllSomatic callset on the HiSeqX data, and the reasons for rejection or no call: not called by any caller (NotCalled), found only in rejected calls of callers (RejectedByCallers), rejected in Panel of Normals filtering step (PanelOfNormal), rejected in common germline filtering step (CommonGermline) or rejected in allele count filtering step (AlleleCount). The lower panels show scatterplots of VAF of the variants in the tumor vs VAF in the normal, VAF in the normal vs depth (DP) at the position in the normal, VAF in the tumor vs depth in the tumor.

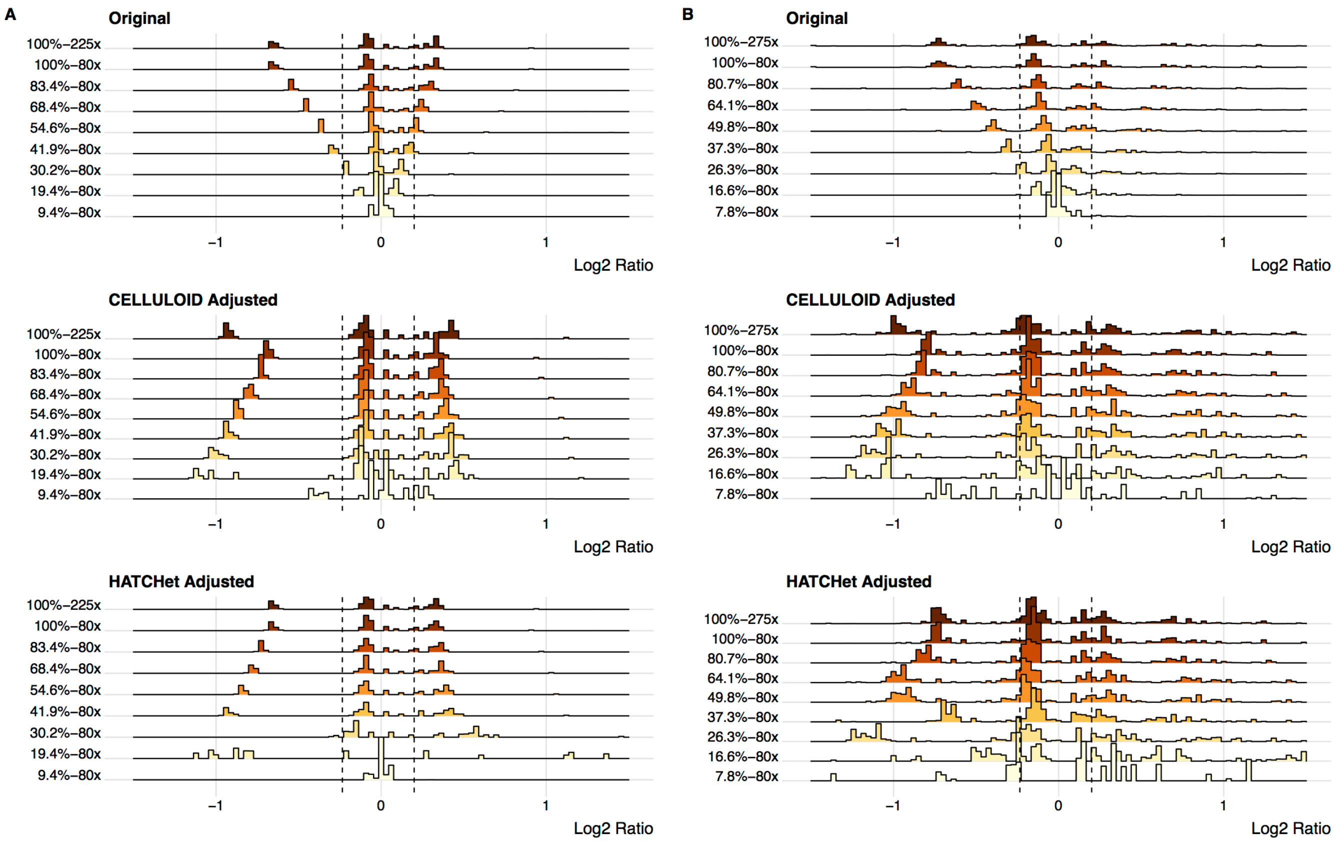

Supplemental Figure S12: Adjustment of Log2 Values in Cell Line Purity Ladder
Density plot showing the log2 values of CNVs called in the purity ladder cell lines for (A) COLO-829 and (B) HCC-1143. The first row shows the original unadjusted log2 values that were called at various purities.

The second row shows the CELLULOID adjusted log2 values at the same purity levels. The third row shows the HATCHet adjusted log2 values at the same purity levels.

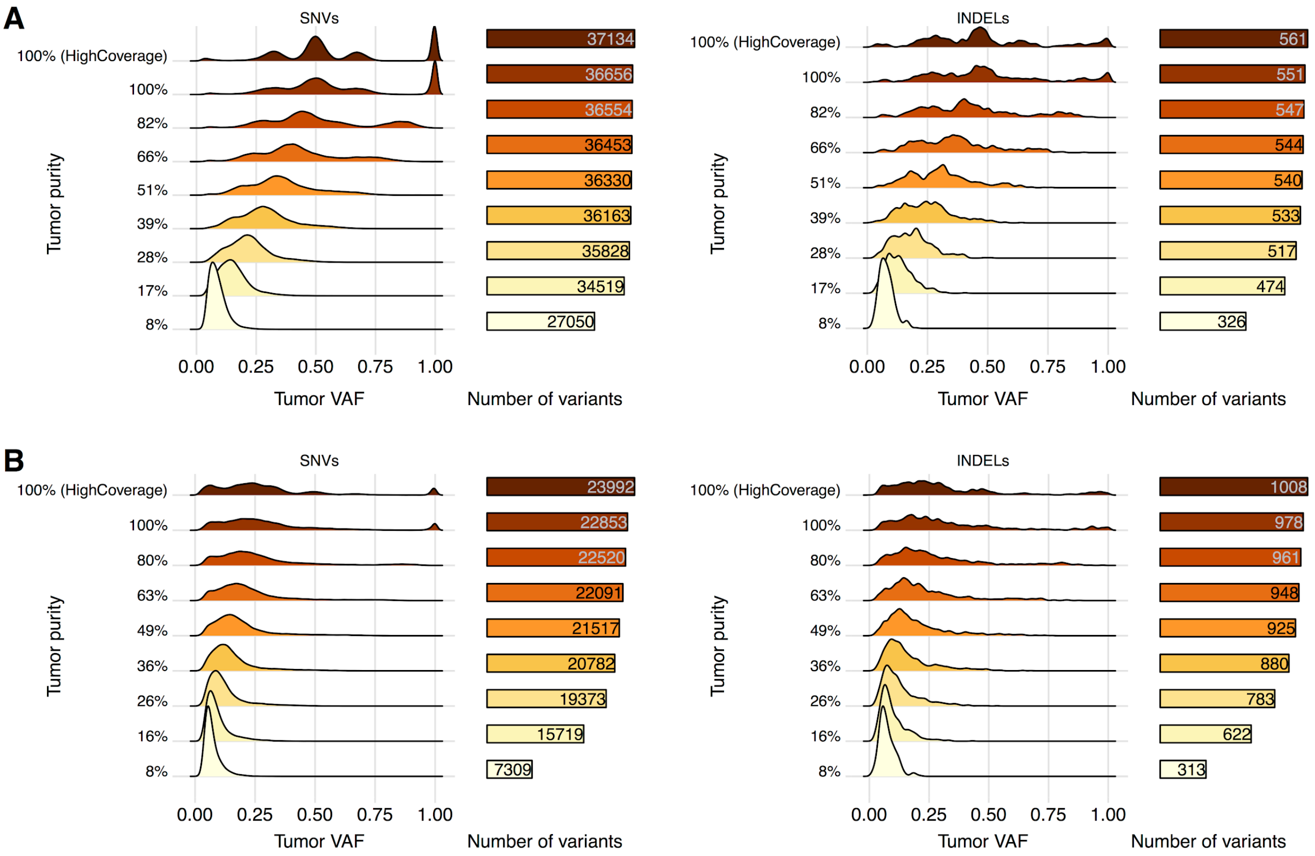

Supplemental Figure S13: Variant allele frequency distribution and number of high confidence SNVs and Indels called in the high coverage data that are also called in the AllSomatic callsets of the purity ladder samples for (A) COLO-829 and (B) HCC-1143.

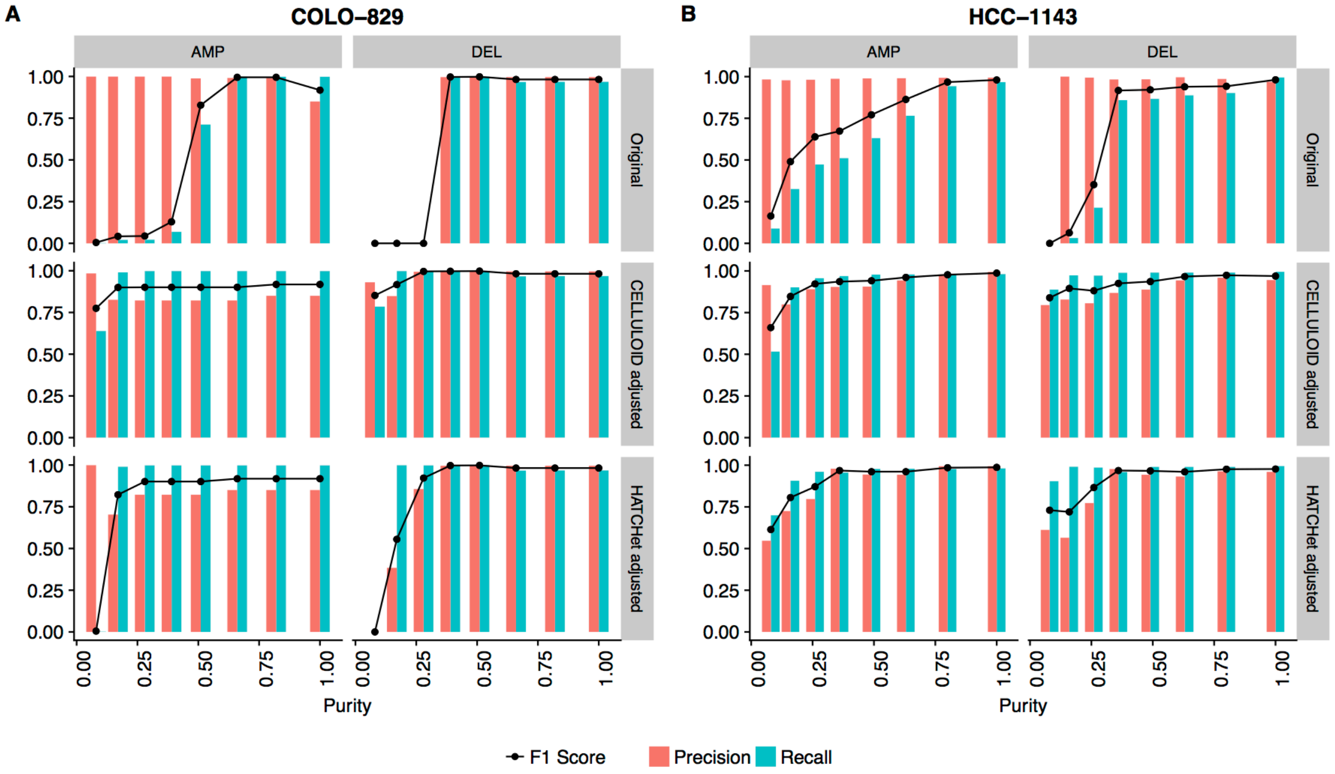

Supplemental Figure S14: Precision, recall and F1 scores at different simulated purities for CNVs without (Original) and with (CELLULOID/HATCHet) adjustments of log2 values for purity and ploidy. Panel A corresponds to COLO-829, Panel B to HCC-1143.

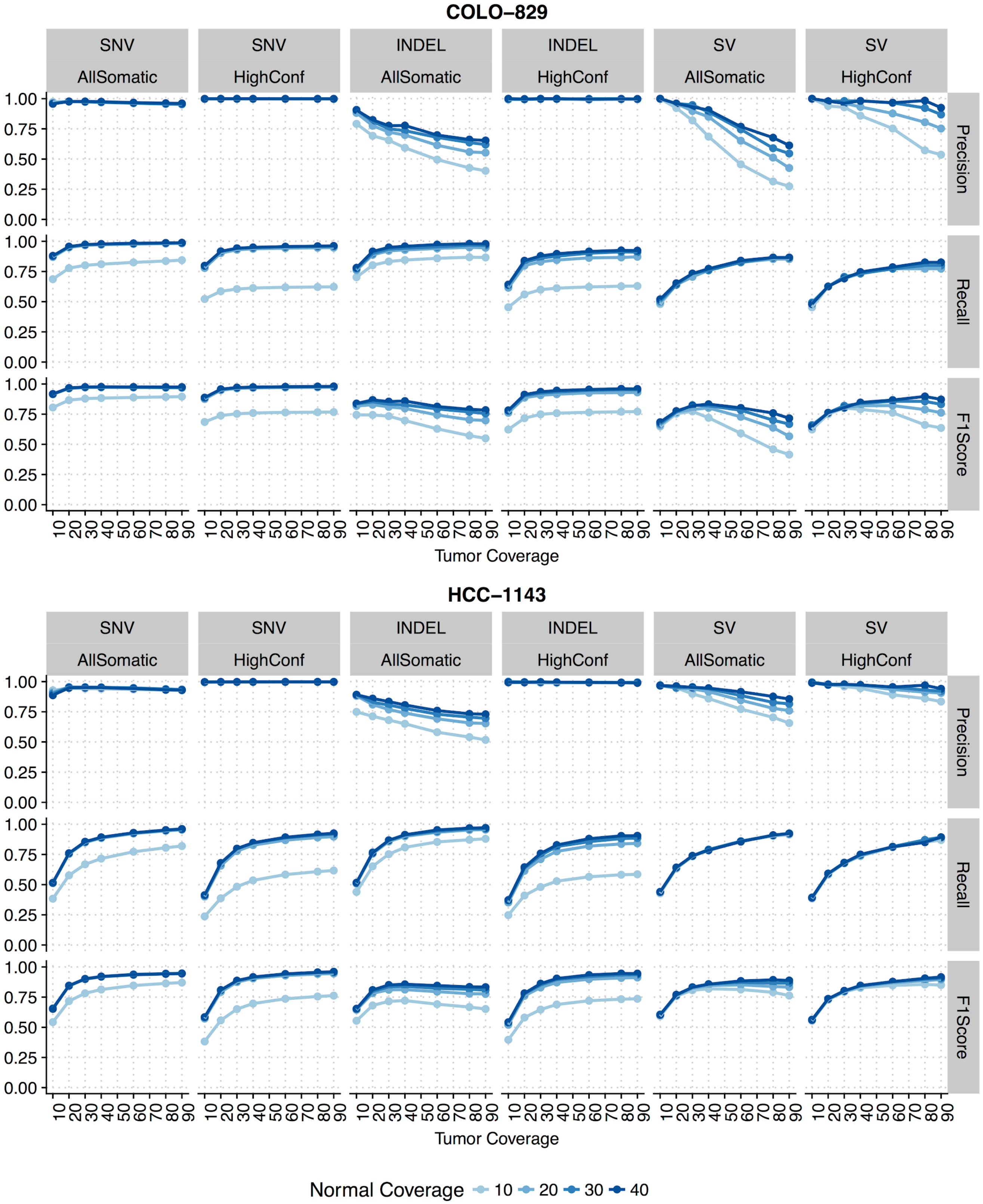

Supplemental Figure S15 Precision, recall and F1 scores for AllSomatic and HighConfidence SNV, INDEL and SV callsets at different coverages of tumor and normal data from COLO-829 (top) and HCC-1143 (bottom)

A

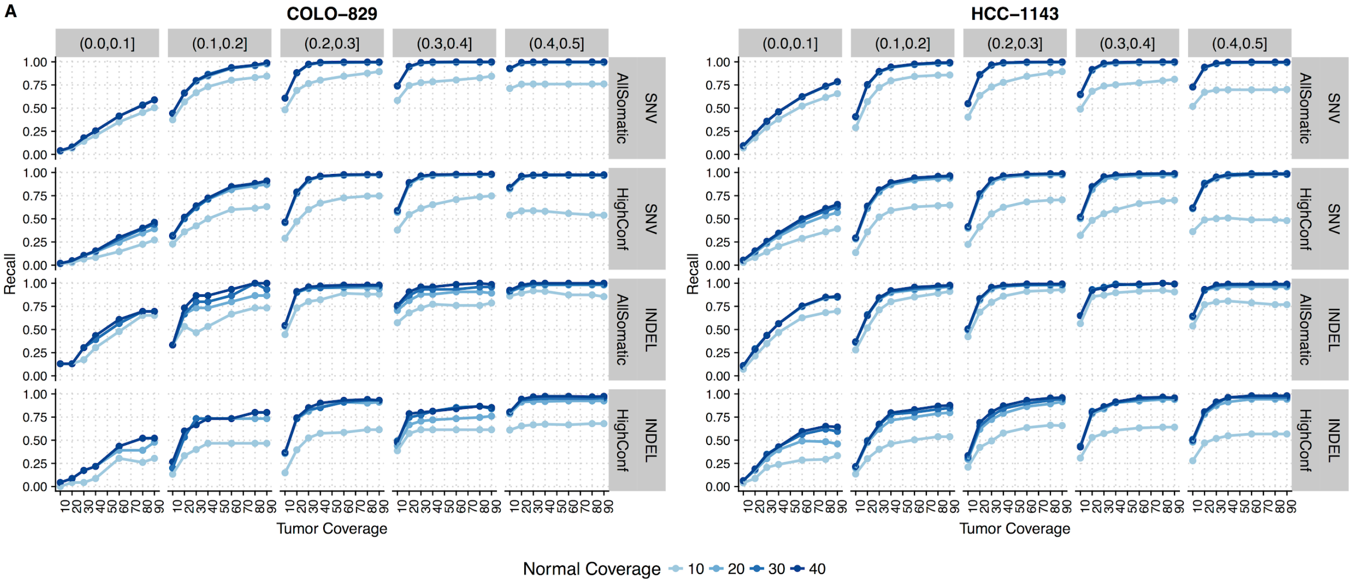

B

	(0.0,0.1]	(0.1,0.2]	(0.2,0.3]	(0.3,0.4]	(0.4,0.5]
SNV	952	342	2271	4807	8282
INDEL	23	15	101	75	159

	(0.0,0.1]	(0.1,0.2]	(0.2,0.3]	(0.3,0.4]	(0.4,0.5]
SNV	4080	5059	6576	3716	1423
INDEL	126	236	272	117	104

Supplemental Figure S16: (A) Recall of SNVs and Indels in different variant allele frequency ranges, for different tumor and normal coverages of COLO-829 (left) and HCC-1143 (right). (B) Number of SNVs and Indels in the truth set (high confidence callset of high coverage data) in the different VAF ranges for COLO-829 (left) and HCC-1143 (right)

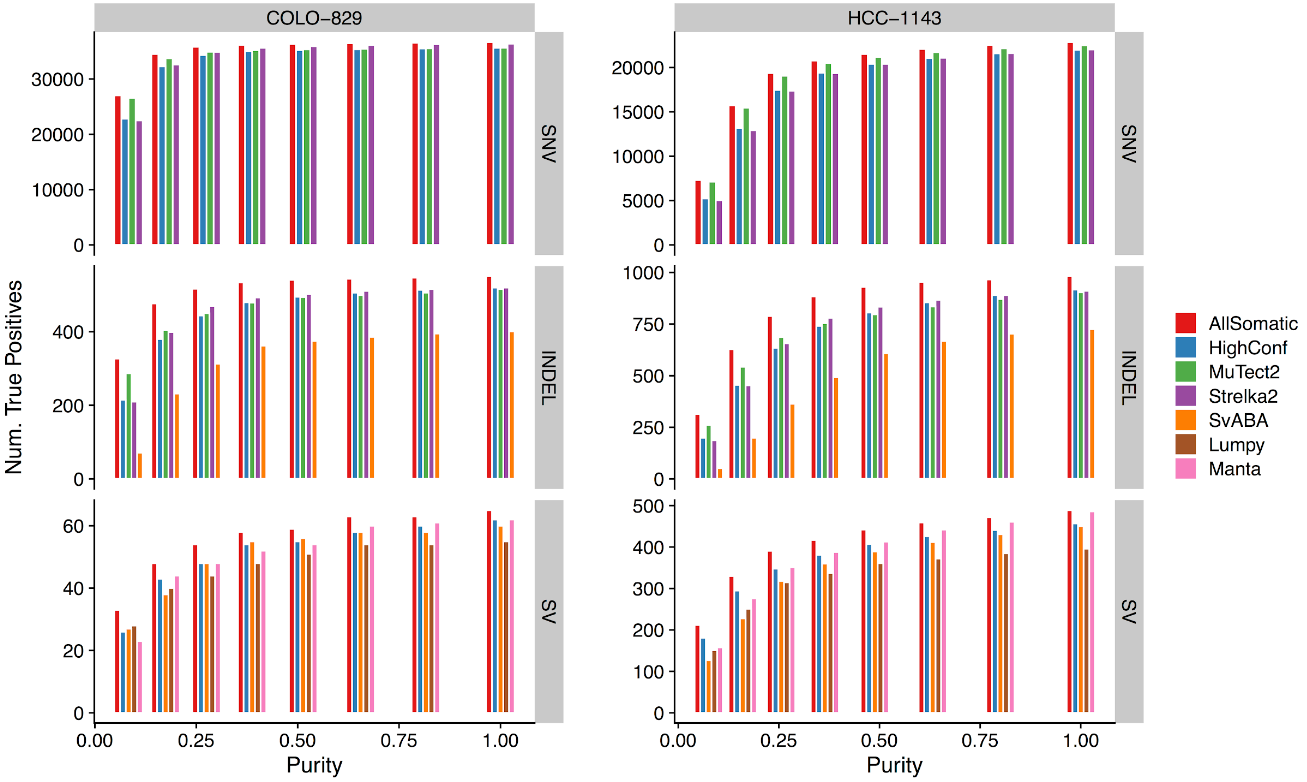

Supplemental Figure S17: Number of true positive variants called on the purity ladder samples in AllSomatic and HighConf callsets of the NYGC pipeline, and by individual callers. We find higher true positive calls in the AllSomatic callset, which combines calls from multiple callers, than any individual caller.

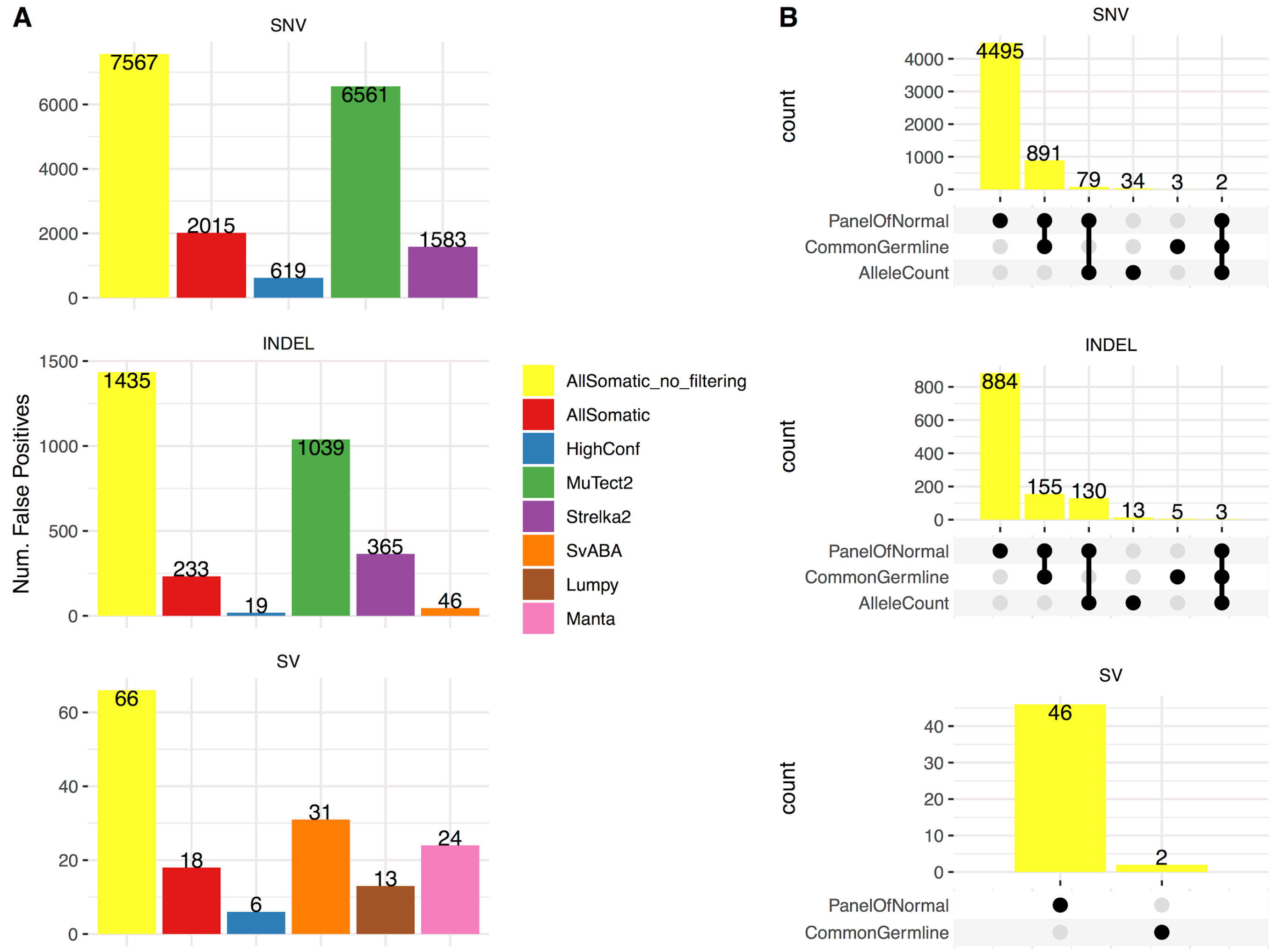

Supplemental Figure S18: (A) Number of calls made when we treated 90X average coverage COLO-829BL (normal) cell line data as “tumor”, and a distinct set of reads from the same cell line at 40X average coverage as “normal”. Since it is the same cell line sample, any variant called on this pairing is a false positive. The yellow bar shows the number of false positive variants called by NYGC pipeline before the NYGC filtering steps (which include panel of normal filtering, common germline filtering and allele counts based filtering (see Methods)). (B) UpSet plots that show the number of variants removed from the AllSomatic callset by each filtering step.
