# Supplementary material for: Deep whole-genome sequencing of 3 cancer cell lines on 2 sequencing platforms
Source: Sci Rep. 2019 Dec 13;9:19123. doi: 10.1038/s41598-019-55636-3 (PMC6911065; doi:10.1038/s41598-019-55636-3)
Supplement: Supplementary file 2 — Supplementary File [file 41598_2019_55636_MOESM2_ESM.zip › pipeline_specs/calling.html]

# Calling

Variant callers are run with aligned BAMs. The output includes calls for SNVs,
INDELs, MNVs, complex variants, SVs, CNVs.

- Taskflow diagram: Calling
- Manta
- Filter non-pass
- Strelka2
- Lancet
- GATK Merge Sort VCF
- Mutect2
- Mutect2 filter
- GATK Merge Sort VCF
- Get discordant
- ExtractSplitReads BwaMem
- Lumpy
- Lumpy filter
- Lumpy split
- SVtyper
- Header blank VCF
- Remove bad ref metadata
- Remove contig
- GATK Merge Sort VCF
- SVtyper filter
- Index VCF
- Unique reads
- Bicseq2 norm
- Bicseq2

# Taskflow diagram: Calling

# STEP: Manta

---

- VERSION:
  'manta 1.4.0'
- REFERENCE FILES:

```
<REFERENCE_FASTA> 
<GRCh38.callregions BED>
```

The reference FASTA GRCh38\_full\_analysis\_set\_plus\_decoy\_hla.fa is available on the 1000genome FTP.

The callregions BED file restricts Manta to the chromosomes to speed up its runtime.

- COMMAND:

```
# Run command...
manta/manta-1.4.0/bin/configManta.py \
--normalBam NORMAL.final.bam \
--tumorBam TUMOR.final.bam \
--referenceFasta <REFERENCE_FASTA>  \
--callRegions <GRCh38.callregions BED> \
--runDir output \
&& \
output/runWorkflow.py \
--mode local \
--job 8 \
--memGb 64 \
```

# STEP: Filter non-pass

---

- VERSION:
  'gatk 4.0.0'
- DEPENDENCIES:

```
java jdk-1.8.0.45
```

- REFERENCE FILES:

```
<REFERENCE_FASTA>
```

The reference FASTA GRCh38\_full\_analysis\_set\_plus\_decoy\_hla.fa is available on the 1000genome FTP.

- COMMAND:

```
# Run command...
gatk/gatk-4.0.0/gatk \
SelectVariants \
--java-options "-Xmx8g -XX:ParallelGCThreads=4" \
-R <REFERENCE_FASTA> \
-V TUMOR--NORMAL.manta.v1.4.0.vcf \
-O TUMOR--NORMAL.manta.v1.4.0.filtered.vcf \
--exclude-filtered
```

# STEP: Strelka2

---

- VERSION:
  'strelka 2.9.3'
- REFERENCE FILES:

```
<REFERENCE_FASTA> 
<GRCh38.callregions BED>
```

The reference FASTA GRCh38\_full\_analysis\_set\_plus\_decoy\_hla.fa is available on the 1000genome FTP.

The callregions BED file restricts Strelka2 to the chromosomes to speed up its runtime.

- COMMAND:

```
# Run command...
strelka/strelka-2.9.3/bin/configureStrelkaSomaticWorkflow.py \
--normalBam NORMAL.final.bam \
--tumorBam TUMOR.final.bam \
--referenceFasta <REFERENCE_FASTA> \
--callRegions <GRCh38.callregions BED> \
--indelCandidates candidateSmallIndels.vcf.gz \
--config configureStrelkaSomaticWorkflow.py.ini \
--runDir output \
&& \
output/runWorkflow.py \
--mode local \
--job 8 \
--memGb 40
```

# STEP: Lancet

---

- VERSION:
  'lancet 1.0.7'
- REFERENCE FILES:

```
<REFERENCE_FASTA> 
<knownGene_hg20_exons_padding_5 BED for chrom i>
```

The FASTA GRCh38\_full\_analysis\_set\_plus\_decoy\_hla.fa is available on the 1000genome FTP.
The knownGene BED file is downloaded from the UCSC table browser and padding of 5 bp is added to each gene.

- COMMAND:

```
# Run command...
lancet/lancet-1.0.7/lancet \
--normal NORMAL.final.bam \
--tumor TUMOR.final.bam \
--bed <knownGene_hg20_exons_padding_5 BED for chrom i> \
--ref <REFERENCE_FASTA>  \
--min-k 11 \
--low-cov 1 \
--min-phred-fisher 5 \
--min-strand-bias 1 \
--min-alt-count-tumor 3 \
--min-vaf-tumor 0.04 \
--num-threads 2 \
> TUMOR--NORMAL_chr1.lancet.v1.0.7.vcf
```

# STEP: GATK Merge Sort VCF

---

- VERSION:
  'gatk 4.0.0'
- DEPENDENCIES:

```
java jdk-1.8.0.45
```

- REFERENCE FILES:

```
<REFERENCE_FASTA_DICT>
```

The reference dictionary for the FASTA GRCh38\_full\_analysis\_set\_plus\_decoy\_hla.fa is available on the 1000genome FTP.

- COMMAND:

```
# Run command...
gatk/gatk-4.0.0/gatk \
SortVcf \
--java-options "-Xmx8196m -XX:ParallelGCThreads=4" \
--TMP_DIR tmp \
-SD <REFERENCE_FASTA_DICT> \
-I TUMOR--NORMAL_chr1.lancet.v1.0.7.vcf \
-I TUMOR--NORMAL_chr2.lancet.v1.0.7.vcf \
-I TUMOR--NORMAL_chr3.lancet.v1.0.7.vcf \
-I TUMOR--NORMAL_chr4.lancet.v1.0.7.vcf \
-I TUMOR--NORMAL_chr5.lancet.v1.0.7.vcf \
-I TUMOR--NORMAL_chr6.lancet.v1.0.7.vcf \
-I TUMOR--NORMAL_chr7.lancet.v1.0.7.vcf \
-I TUMOR--NORMAL_chr8.lancet.v1.0.7.vcf \
-I TUMOR--NORMAL_chr9.lancet.v1.0.7.vcf \
-I TUMOR--NORMAL_chr10.lancet.v1.0.7.vcf \
-I TUMOR--NORMAL_chr11.lancet.v1.0.7.vcf \
-I TUMOR--NORMAL_chr12.lancet.v1.0.7.vcf \
-I TUMOR--NORMAL_chr13.lancet.v1.0.7.vcf \
-I TUMOR--NORMAL_chr14.lancet.v1.0.7.vcf \
-I TUMOR--NORMAL_chr15.lancet.v1.0.7.vcf \
-I TUMOR--NORMAL_chr16.lancet.v1.0.7.vcf \
-I TUMOR--NORMAL_chr17.lancet.v1.0.7.vcf \
-I TUMOR--NORMAL_chr18.lancet.v1.0.7.vcf \
-I TUMOR--NORMAL_chr19.lancet.v1.0.7.vcf \
-I TUMOR--NORMAL_chr20.lancet.v1.0.7.vcf \
-I TUMOR--NORMAL_chr21.lancet.v1.0.7.vcf \
-I TUMOR--NORMAL_chr22.lancet.v1.0.7.vcf \
-I TUMOR--NORMAL_chrX.lancet.v1.0.7.vcf \
-O TUMOR--NORMAL.lancet.v1.0.7.sorted.vcf
```

# STEP: Mutect2

---

- VERSION:
  'gatk 4.0.5.1'
- DEPENDENCIES:

```
java jdk-1.8.0.45
```

- REFERENCE FILES:

```
<REFERENCE_FASTA>
```

The reference FASTA GRCh38\_full\_analysis\_set\_plus\_decoy\_hla.fa is available on the 1000genome FTP.

- COMMAND:

```
# Run command...
gatk/gatk-4.0.5.1/gatk \
Mutect2 \
--java-options "-Xmx8196m -XX:ParallelGCThreads=4" \
--TMP_DIR tmp \
--reference <REFERENCE_FASTA> \
-L chr1 \
-I TUMOR.final.bam \
-I NORMAL.final.bam \
-tumor TUMOR \
-normal NORMAL \
-O TUMOR--NORMAL_chr1.mutect2.v4.0.5.1.raw.vcf
```

# STEP: Mutect2 filter

---

- VERSION:
  'gatk 4.0.5.1'
- DEPENDENCIES:

```
java jdk-1.8.0.45
```

- REFERENCE FILES:

```
<REFERENCE_FASTA>
```

The reference FASTA GRCh38\_full\_analysis\_set\_plus\_decoy\_hla.fa is available on the 1000genome FTP.

- COMMAND:

```
# Run command...
gatk/gatk-4.0.5.1/gatk \
FilterMutectCalls \
--java-options "-Xmx8196m -XX:ParallelGCThreads=4" \
--TMP_DIR tmp \
--reference <REFERENCE_FASTA>  \
-V TUMOR--NORMAL_chr1.mutect2.v4.0.5.1.raw.vcf \
-O TUMOR--NORMAL_chr1.mutect2.v4.0.5.1.vcf
```

# STEP: GATK Merge Sort VCF

---

- VERSION:
  'gatk 4.0.0'
- DEPENDENCIES:

```
java jdk-1.8.0.45
```

- REFERENCE FILES:

```
<REFERENCE_FASTA_DICT>
```

The reference dictionary for the FASTA GRCh38\_full\_analysis\_set\_plus\_decoy\_hla.fa is available on the 1000genome FTP.

- COMMAND:

```
# Run command...
gatk/gatk-4.0.0/gatk \
SortVcf \
--java-options "-Xmx8196m -XX:ParallelGCThreads=4" \
--TMP_DIR tmp \
-SD <REFERENCE_FASTA_DICT> \
-I TUMOR--NORMAL_chr1.mutect2.v4.0.5.1.vcf \
-I TUMOR--NORMAL_chr2.mutect2.v4.0.5.1.vcf \
-I TUMOR--NORMAL_chr3.mutect2.v4.0.5.1.vcf \
-I TUMOR--NORMAL_chr4.mutect2.v4.0.5.1.vcf \
-I TUMOR--NORMAL_chr5.mutect2.v4.0.5.1.vcf \
-I TUMOR--NORMAL_chr6.mutect2.v4.0.5.1.vcf \
-I TUMOR--NORMAL_chr7.mutect2.v4.0.5.1.vcf \
-I TUMOR--NORMAL_chr8.mutect2.v4.0.5.1.vcf \
-I TUMOR--NORMAL_chr9.mutect2.v4.0.5.1.vcf \
-I TUMOR--NORMAL_chr10.mutect2.v4.0.5.1.vcf \
-I TUMOR--NORMAL_chr11.mutect2.v4.0.5.1.vcf \
-I TUMOR--NORMAL_chr12.mutect2.v4.0.5.1.vcf \
-I TUMOR--NORMAL_chr13.mutect2.v4.0.5.1.vcf \
-I TUMOR--NORMAL_chr14.mutect2.v4.0.5.1.vcf \
-I TUMOR--NORMAL_chr15.mutect2.v4.0.5.1.vcf \
-I TUMOR--NORMAL_chr16.mutect2.v4.0.5.1.vcf \
-I TUMOR--NORMAL_chr17.mutect2.v4.0.5.1.vcf \
-I TUMOR--NORMAL_chr18.mutect2.v4.0.5.1.vcf \
-I TUMOR--NORMAL_chr19.mutect2.v4.0.5.1.vcf \
-I TUMOR--NORMAL_chr20.mutect2.v4.0.5.1.vcf \
-I TUMOR--NORMAL_chr21.mutect2.v4.0.5.1.vcf \
-I TUMOR--NORMAL_chr22.mutect2.v4.0.5.1.vcf \
-I TUMOR--NORMAL_chrX.mutect2.v4.0.5.1.vcf \
-O TUMOR--NORMAL.mutect2.v4.0.5.1.sorted.vcf
```

# STEP: Get discordant

---

- VERSION:
  'samtools 1.4.1'
- COMMAND:

```
# Run command...
samtools/samtools-1.4.1/bin/samtools view \
-h \
-b \
-F 1294 \
--threads 4 \
TUMOR.final.bam \
| samtools/samtools-1.4.1/bin/samtools \
sort \
--threads 4 \
-m 140G \
-o TUMOR_discordant.bam
```

# STEP: ExtractSplitReads BwaMem

---

- VERSION:
  'extractSplitReads\_BwaMem 0.1.0'
- DEPENDENCIES:

```
samtools 1.4.1
```

- COMMAND:

```
# Run command...
samtools view \
-h \
--threads 4 \
TUMOR.final.bam \
| extractSplitReads_BwaMem \
-i stdin \
| samtools \
view \
-b -h \
--threads 4 \
| samtools \
sort \
--threads 4 \
-m 140G \
-o TUMOR_split_read.bam
```

# STEP: Lumpy

---

- VERSION:
  'lumpy 0.2.13'
- REFERENCE FILES:

```
<exclude GRCh38_contigs BED>
```

Exclude BED is a list of contigs to skip in the reference. It includes everthing except the chromosomes.

- COMMAND:

```
# Run command...
lumpy/lumpy-0.2.13/bin/lumpy \
-mw 4 \
-tt 0.0 \
-t TUMOR--NORMAL \
-x <exclude GRCh38_contigs BED> \
-sr bam_file:TUMOR_split_read.bam,back_distance:10,weight:1,id:TUMOR,min_mapping_threshold:20 \
-pe bam_file:TUMOR_discordant.bam,histo_file:TUMOR_paired_end.histo,mean:424.579395274,stdev:116.065902644,read_length:151,min_non_overlap:151,discordant_z:5,back_distance:10,weight:1,id:TUMOR,min_mapping_threshold:20 \
-sr bam_file:NORMAL_split_read.bam,back_distance:10,weight:1,id:NORMAL,min_mapping_threshold:20 \
-pe bam_file:NORMAL_discordant.bam,histo_file:NORMAL_paired_end.histo,mean:424.579395274,stdev:116.065902644,read_length:151,min_non_overlap:151,discordant_z:5,back_distance:10,weight:1,id:NORMAL,min_mapping_threshold:20 \
> TUMOR--NORMAL.lumpy.v0.2.13.vcf
```

# STEP: Lumpy filter

---

In house script that filters calls with fewer than 2 paired-end supporting reads. Each
passing call must also have no paired-end or split-read support in the normal.

- VERSION:
  '1.0'
- DEPENDENCIES:

```
vcftools 0.1.14
```

- COMMAND:

```
# Run command...
perl/perl-5.22.0/bin/perl \
lumpy_filter.pl \
-in TUMOR--NORMAL.lumpy.v0.2.13.vcf \
-out TUMOR--NORMAL.lumpy.v0.2.13.filtered.vcf \
-tumor TUMOR \
-normal NORMAL
```

# STEP: Lumpy split

---

In-house script to divide input while keeping breakpoints from the same SV together.
Script uses a list of chromosomes and there lengths as metadata.

- DEPENDENCIES:

```
pysam >= 0.11.2.1
```

- COMMAND:

```
# Run command...
python \
svtyper_split.py \
TUMOR--NORMAL.lumpy.v0.2.13.filtered.vcf \
TUMOR--NORMAL.lumpy.v0.2.13.filtered \
chrom_lengths.txt
```

# STEP: SVtyper

---

- VERSION:
  'svtyper 0.6.1'
- DEPENDENCIES:

```
perl 5.22.0
```

- COMMAND:

```
# Run command...
svtyper/svtyper-0.6.1/bin/svtyper \
-i TUMOR--NORMAL.lumpy.v0.2.13.filtered.chr1.vcf \
-B NORMAL.final.bam \
-o TUMOR--NORMAL.lumpy.v0.2.13.filtered.typed.chr1.vcf
```

# STEP: Header blank VCF

---

In-house script to add header to VCF so that it is valid and can be merged by standard tools.

- DEPENDENCIES:

```
pysam >= 0.11.2.1
```

- COMMAND:

```
# Run command...
python \
header_blank_vcf.py \
TUMOR--NORMAL.lumpy.v0.2.13.filtered.typed.chr1.vcf \
TUMOR--NORMAL.lumpy.v0.2.13.filtered.vcf \
TUMOR--NORMAL.lumpy.v0.2.13.filtered.typed.chr1.vcf
```

# STEP: Remove bad ref metadata

---

In-house script to print a VCF file skipping empty ##reference= descriptions.
This is step prevents GATK from throwing an error.

- COMMAND:

```
# Run command...
python \
remove_bad_ref_meta.py \
TUMOR--NORMAL.lumpy.v0.2.13.filtered.typed.chr1.vcf \
TUMOR--NORMAL.lumpy.v0.2.13.filtered.typed.chr1.vcf
```

# STEP: Remove contig

---

In house script to remove contig lines from the VCF header.
This is step prevents GATK from throwing an error if contig lines in the VCF header are not in the same order as the FASTA dictionary file or if they are missing the required length field.

- COMMAND:

```
# Run command...
python \
remove_contig.py \
TUMOR--NORMAL.lumpy.v0.2.13.filtered.typed.chr1.vcf \
TUMOR--NORMAL.lumpy.v0.2.13.filtered.typed.chr1.vcf
```

# STEP: GATK Merge Sort VCF

---

- VERSION:
  'gatk 4.0.0'
- DEPENDENCIES:

```
java jdk-1.8.0.45
```

- REFERENCE FILES:

```
<REFERENCE_FASTA_DICT>
```

The reference FASTA GRCh38\_full\_analysis\_set\_plus\_decoy\_hla.fa is available on the 1000genome FTP.
The .dict file is derived from this FASTA.

- COMMAND:

```
# Run command...
gatk/gatk-4.0.0/gatk \
SortVcf \
--java-options "-Xmx8196m -XX:ParallelGCThreads=4" \
--TMP_DIR tmp \
-SD <REFERENCE_FASTA_DICT>  \
-I TUMOR--NORMAL.lumpy.v0.2.13.filtered.typed.chr1.vcf \
-I TUMOR--NORMAL.lumpy.v0.2.13.filtered.typed.chr2.vcf \
-I TUMOR--NORMAL.lumpy.v0.2.13.filtered.typed.chr3.vcf \
-I TUMOR--NORMAL.lumpy.v0.2.13.filtered.typed.chr4.vcf \
-I TUMOR--NORMAL.lumpy.v0.2.13.filtered.typed.chr5.vcf \
-I TUMOR--NORMAL.lumpy.v0.2.13.filtered.typed.chr6.vcf \
-I TUMOR--NORMAL.lumpy.v0.2.13.filtered.typed.chr7.vcf \
-I TUMOR--NORMAL.lumpy.v0.2.13.filtered.typed.chr8.vcf \
-I TUMOR--NORMAL.lumpy.v0.2.13.filtered.typed.chr9.vcf \
-I TUMOR--NORMAL.lumpy.v0.2.13.filtered.typed.chr10.vcf \
-I TUMOR--NORMAL.lumpy.v0.2.13.filtered.typed.chr11.vcf \
-I TUMOR--NORMAL.lumpy.v0.2.13.filtered.typed.chr12.vcf \
-I TUMOR--NORMAL.lumpy.v0.2.13.filtered.typed.chr13.vcf \
-I TUMOR--NORMAL.lumpy.v0.2.13.filtered.typed.chr14.vcf \
-I TUMOR--NORMAL.lumpy.v0.2.13.filtered.typed.chr15.vcf \
-I TUMOR--NORMAL.lumpy.v0.2.13.filtered.typed.chr16.vcf \
-I TUMOR--NORMAL.lumpy.v0.2.13.filtered.typed.chr17.vcf \
-I TUMOR--NORMAL.lumpy.v0.2.13.filtered.typed.chr18.vcf \
-I TUMOR--NORMAL.lumpy.v0.2.13.filtered.typed.chr19.vcf \
-I TUMOR--NORMAL.lumpy.v0.2.13.filtered.typed.chr20.vcf \
-I TUMOR--NORMAL.lumpy.v0.2.13.filtered.typed.chr21.vcf \
-I TUMOR--NORMAL.lumpy.v0.2.13.filtered.typed.chr22.vcf \
-I TUMOR--NORMAL.lumpy.v0.2.13.filtered.typed.chrX.vcf \
-I TUMOR--NORMAL.lumpy.v0.2.13.filtered.typed.chrY.vcf \
-O TUMOR--NORMAL.lumpy.v0.2.13.svtyper.v0.6.1.sorted.vcf
```

# STEP: SVtyper filter

---

In-house script that removes variants if normal 'AO' is 0 and tumor
'AO' is greater than 0.

- COMMAND:

```
# Run command...
python \
svtyper_filter.py \
TUMOR--NORMAL.lumpy.v0.2.13.svtyper.v0.6.1.vcf \
TUMOR--NORMAL.lumpy.v0.2.13.svtyper.v0.6.1.filtered.vcf \
TUMOR \
NORMAL
```

# STEP: Index VCF

---

- VERSION:
  'gatk 4.0.0'
- DEPENDENCIES:

```
java jdk-1.8.0.45
```

- COMMAND:

```
# Run command...
gatk/gatk-4.0.0/gatk \
IndexFeatureFile \
--java-options "-Xmx8g -XX:ParallelGCThreads=4" \
-F TUMOR--NORMAL.lumpy.v0.2.13.svtyper.v0.6.1.filtered.vcf
```

# STEP: Unique reads

---

- VERSION:
  'bicseq2 0.2.6'
- COMMAND:

```
# Run command...
bicseq2/bicseq2-0.2.6/samtools \
view \
-U BWA,TUMOR/TUMOR_,N,N \
TUMOR.final.bam \
&& \
bicseq2/bicseq2-0.2.6/samtools \
view \
-U BWA,NORMAL/NORMAL_,N,N \
NORMAL.final.bam
```

# STEP: Bicseq2 norm

---

- VERSION:
  'bicseq2 0.2.6'
- COMMAND:

```
# Run command...
perl \
bicseq2/bicseq2-0.2.6/BICseq2-norm.pl \
-l=151 \
-s=412 \
-fig=TUMOR.GCvsRD.pdf \
-tmp=TUMOR/ \
TUMORbicseq2.config \
TUMOR.params.out
```

# STEP: Bicseq2

---

- VERSION:
  'bicseq2 0.2.6'
- COMMAND:

```
# Run command...
perl \
bicseq2/bicseq2-0.2.6/NBICseq-seg.pl \
--control \
--fig=TUMOR--NORMAL.bicseq2.v0.2.6.png \
--title=TUMOR--NORMAL \
--tmp=tmp \
--lambda=4 \
TUMOR--NORMALbicseq2.seg.config \
TUMOR--NORMAL.bicseq2.v0.2.6.txt
```

---

Published from calling.md
using Pweave 0.30.3
on 08-11-2019.
