# Supplementary material for: Deep whole-genome sequencing of 3 cancer cell lines on 2 sequencing platforms
Source: Sci Rep. 2019 Dec 13;9:19123. doi: 10.1038/s41598-019-55636-3 (PMC6911065; doi:10.1038/s41598-019-55636-3)
Supplement: Supplementary file 2 — Supplementary File [file 41598_2019_55636_MOESM2_ESM.zip › pipeline_specs/pre-processing.html]

# Pre-processing

Multiple FASTQ files (one for each flowcell) are processed in parallel for alignment,
short-alignment marking and fixmate. These BAM files are merged and base quality
scores are recalibrated.

- Taskflow diagram: Pre-processing
- BWA mem
- Short-alignment marking
- BWA fixmate
- Novosort duplicate marking/sorting
- Index BAM
- GATK Downsample
- GATK BSQR
- GATK ApplyBQSR

# Taskflow diagram: Pre-processing

# STEP: BWA mem

---

- VERSION:
  'bwa 0.7.15'
- DEPENDENCIES:

```
samtools-1.3.1
```

- REFERENCE FILES:

```
<REFERENCE_FASTA>
```

The reference FASTA GRCh38\_full\_analysis\_set\_plus\_decoy\_hla.fa is available on the 1000genome FTP

- COMMAND:

```
# Run command...
bwa \
mem \
-Y \
-K 100000000 \
-t 16 \
-R '@RG\tID:<RG_ID>\tPL:<Platform>\tPM:<PlatformModel>\tLB:<SAMPLE>\tDS:<Reference_Build>\tSM:<SAMPLE>\tCN:NYGenome\tPU:<PU>' \
<REFERENCE_FASTA> \
<RG_ID>.R1.fastq.gz \
<RG_ID>.R2.fastq.gz \
| samtools view \
-Shb \
-o <RG_ID>.readgroup.bam \
-
```

# STEP: Short-alignment marking

---

- VERSION:
  'filter\_bam 2.1'

Filter bam is available at nygc-short-alignment-marking.

- DEPENDENCIES:

```
samtools-1.3.1
```

- COMMAND:

```
# Run command...
filter_bam \
-I <RG_ID>.readgroup.bam \
-A1 30 \
-A2 30 \
-o <RG_ID>.readgroup_mark.bam \
| samtools view \
-b \
-o <RG_ID>.readgroup_mark.bam \
-
```

# STEP: BWA fixmate

---

- VERSION:
  'gatk 4.1.0'
- DEPENDENCIES:

```
java jdk-1.8.0.45
```

- COMMAND:

```
# Run command...
gatk \
FixMateInformation \
--java-options "-Xmx24576m -XX:ParallelGCThreads=1" \
--TMP_DIR <TEMP_DIR> \
--MAX_RECORDS_IN_RAM 2000000 \
--VALIDATION_STRINGENCY SILENT \
--ADD_MATE_CIGAR true \
--ASSUME_SORTED true \
-I <RG_ID>.readgroup_mark.bam \
-O <RG_ID>.readgroup_fixmate.bam
```

# STEP: Novosort duplicate marking/sorting

---

- VERSION:
  'novoalign 3.08.02'
- COMMAND:

```
# Run command...
novosort \
-c 10 \
-m 49152M \
-t temp/ \
-i \
-o <SAMPLE>.merged_dedup.bam \
--forcesort \
--markDuplicates \
<RG_ID_1>.readgroup_fixmate.bam \
<RG_ID_2>.readgroup_fixmate.bam \
2> SAMPLE.novosort_dedup.log
```

# STEP: Index BAM

---

- VERSION:
  'samtools 1.3.1'
- COMMAND:

```
# Run command...
samtools index \
<SAMPLE>.merged_dedup.bam
```

# STEP: GATK Downsample

---

- VERSION:
  'gatk 4.1.0'
- COMMAND:

```
# Run command...
gatk \
DownsampleSam \
--java-options "-Xmx80g -XX:ParallelGCThreads=1" \
--TMP_DIR temp/ \
--STRATEGY Chained \
--RANDOM_SEED 1 \
--CREATE_INDEX \
--MAX_RECORDS_IN_RAM 2000000 \
--VALIDATION_STRINGENCY SILENT \
-P 0.1 \
-I <SAMPLE>.merged_dedup.bam \
-O <SAMPLE>.merged_dedup_10_percent.bam
```

# STEP: GATK BSQR

---

- VERSION:
  'gatk 4.1.0'
- REFERENCE FILES:

```
<REFERENCE_FASTA>
<GATKBUNDLE_Mills_and_1000G_gold_standard>.vcf.gz
<GATKBUNDLE_Known_indels>.vcf.gz
<GATKBUNDLE_dbsnp>.vcf
```

The reference FASTA GRCh38\_full\_analysis\_set\_plus\_decoy\_hla.fa is available on the 1000genome FTP.
The hg38 GATK Bundle is available in several ways (see download instructions)

- COMMAND:

```
# Run command...
gatk \
BaseRecalibrator \
--java-options "-Xmx24576m -XX:ParallelGCThreads=1" \
--tmp-dir temp/ \
-L <AUTOSOMES> \
-I SAMPLE.merged_dedup_10_percent.bam \
-O SAMPLE.recal_data.grp \
--known-sites <GATKBUNDLE_Mills_and_1000G_gold_standard>.vcf.gz \
--known-sites <GATKBUNDLE_Known_indels>.vcf.gz \
--known-sites <GATKBUNDLE_dbsnp>.vcf
```

# STEP: GATK ApplyBQSR

---

- VERSION:
  'gatk 4.1.0'
- REFERENCE FILES:

```
<REFERENCE_FASTA>
```

The reference FASTA GRCh38\_full\_analysis\_set\_plus\_decoy\_hla.fa is available on the 1000genome FTP

- COMMAND:

```
# Run command...
gatk \
ApplyBQSR \
--java-options "-Xmx24576m -XX:ParallelGCThreads=1" \
--tmp-dir temp/ \
-R <REFERENCE_FASTA> \
-I <SAMPLE>.merged_dedup.bam \
-O <SAMPLE>.final.bam \
--bqsr-recal-file <SAMPLE>.recal_data.grp
```

---

Published from pre-processing.md
using Pweave 0.30.3
on 08-11-2019.
