# Supplementary material for: Deep whole-genome sequencing of 3 cancer cell lines on 2 sequencing platforms
Source: Sci Rep. 2019 Dec 13;9:19123. doi: 10.1038/s41598-019-55636-3 (PMC6911065; doi:10.1038/s41598-019-55636-3)
Supplement: Supplementary file 2 — Supplementary File [file 41598_2019_55636_MOESM2_ESM.zip › pipeline_specs/README.html]

# Pipeline specs

Pipeline diagrams and commands for the NYGC v6 somatic pipeline.

## Workflows sections

- Pre-processing Workflow
- Calling Workflow

---

Published from README.md
using Pweave 0.30.3
on 08-11-2019.
