# Supplementary figures and images for: Deep whole-genome sequencing of 3 cancer cell lines on 2 sequencing platforms
Source: Sci Rep. 2019 Dec 13;9:19123. doi: 10.1038/s41598-019-55636-3 (PMC6911065; doi:10.1038/s41598-019-55636-3)

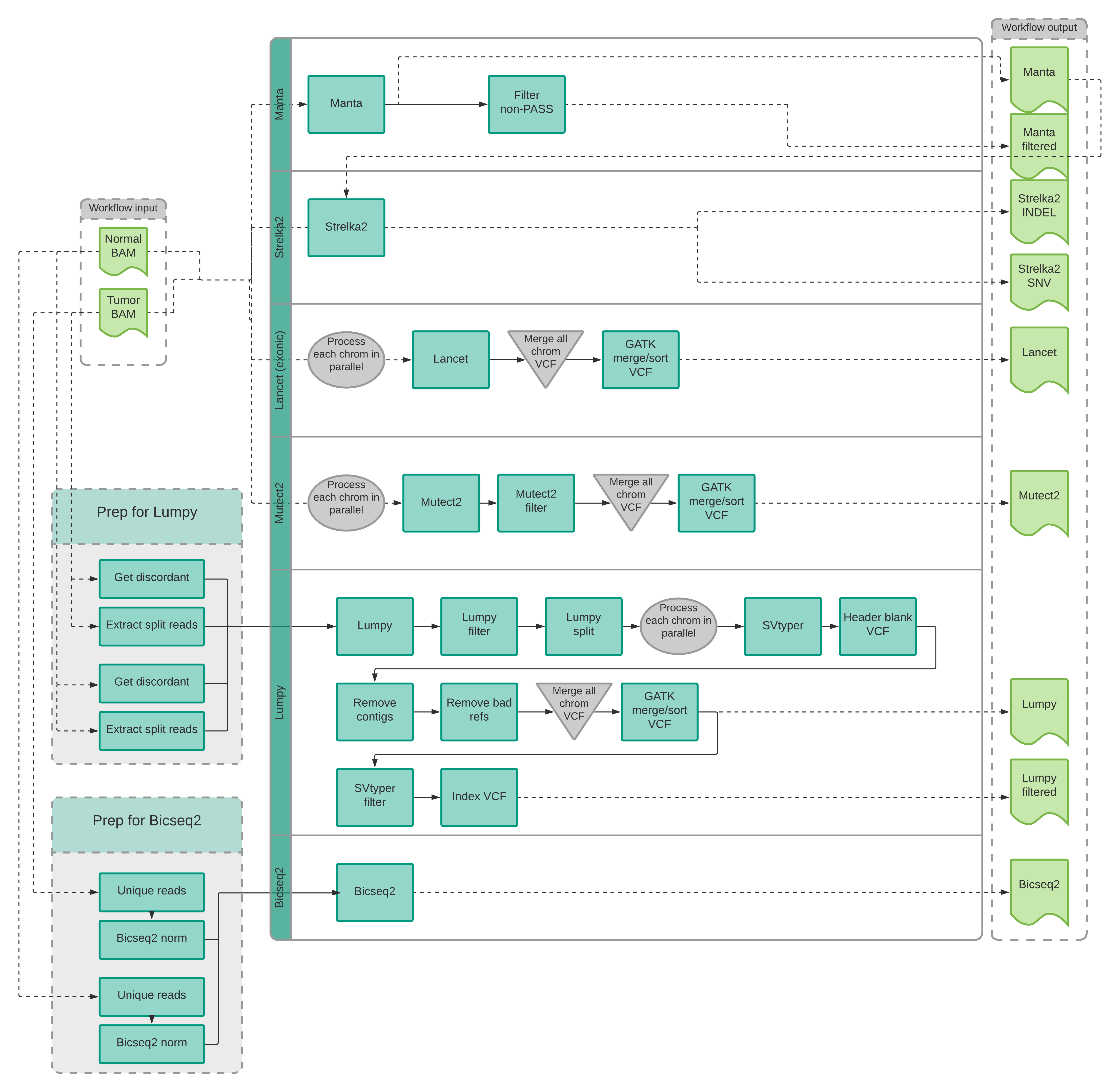

Supplement: Supplementary file 2 — Supplementary File [file 41598_2019_55636_MOESM2_ESM.zip › pipeline_specs/figs/WGS_calling.png]

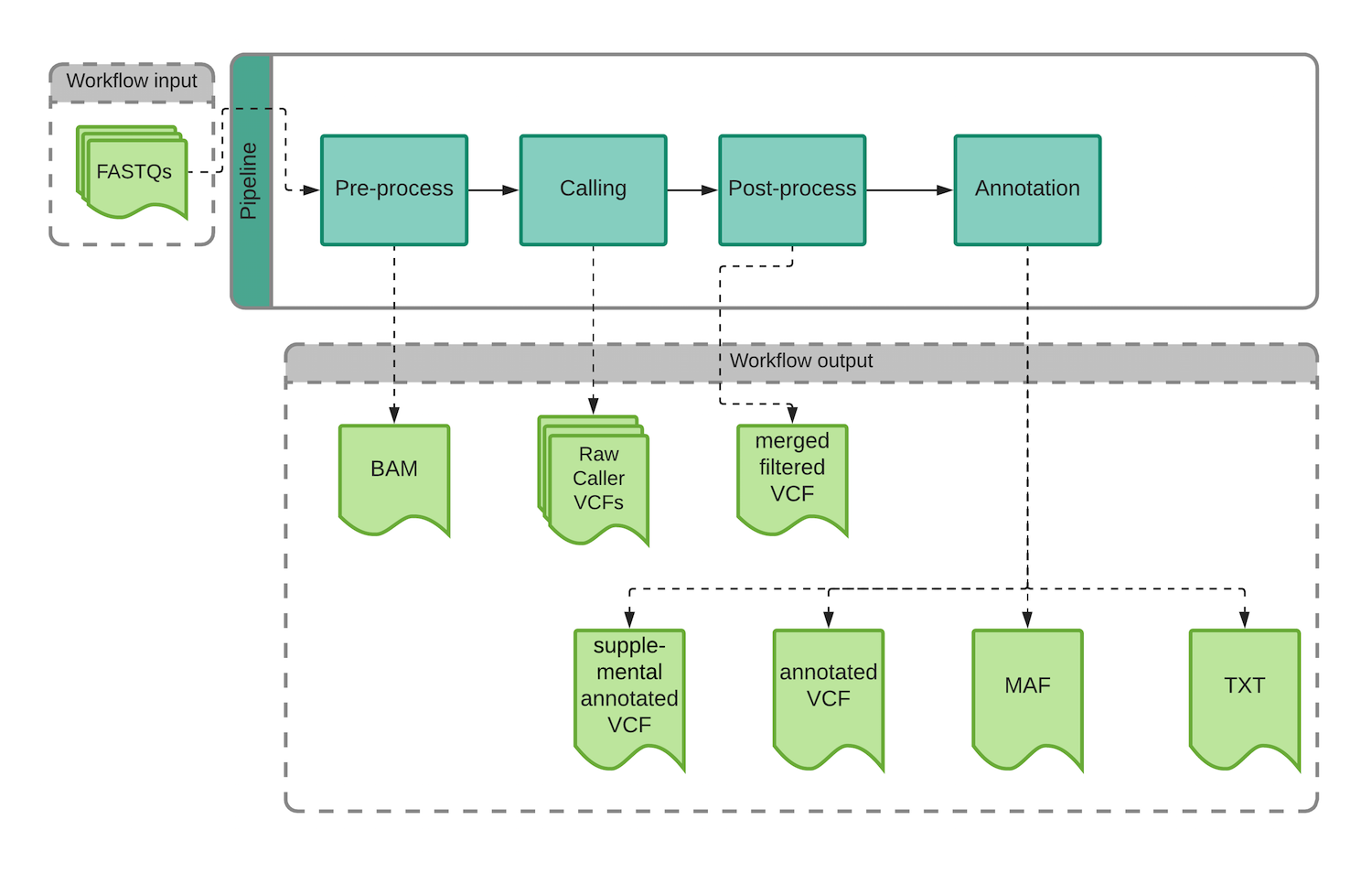

Supplement: Supplementary file 2 — Supplementary File [file 41598_2019_55636_MOESM2_ESM.zip › pipeline_specs/figs/Pipeline.png]

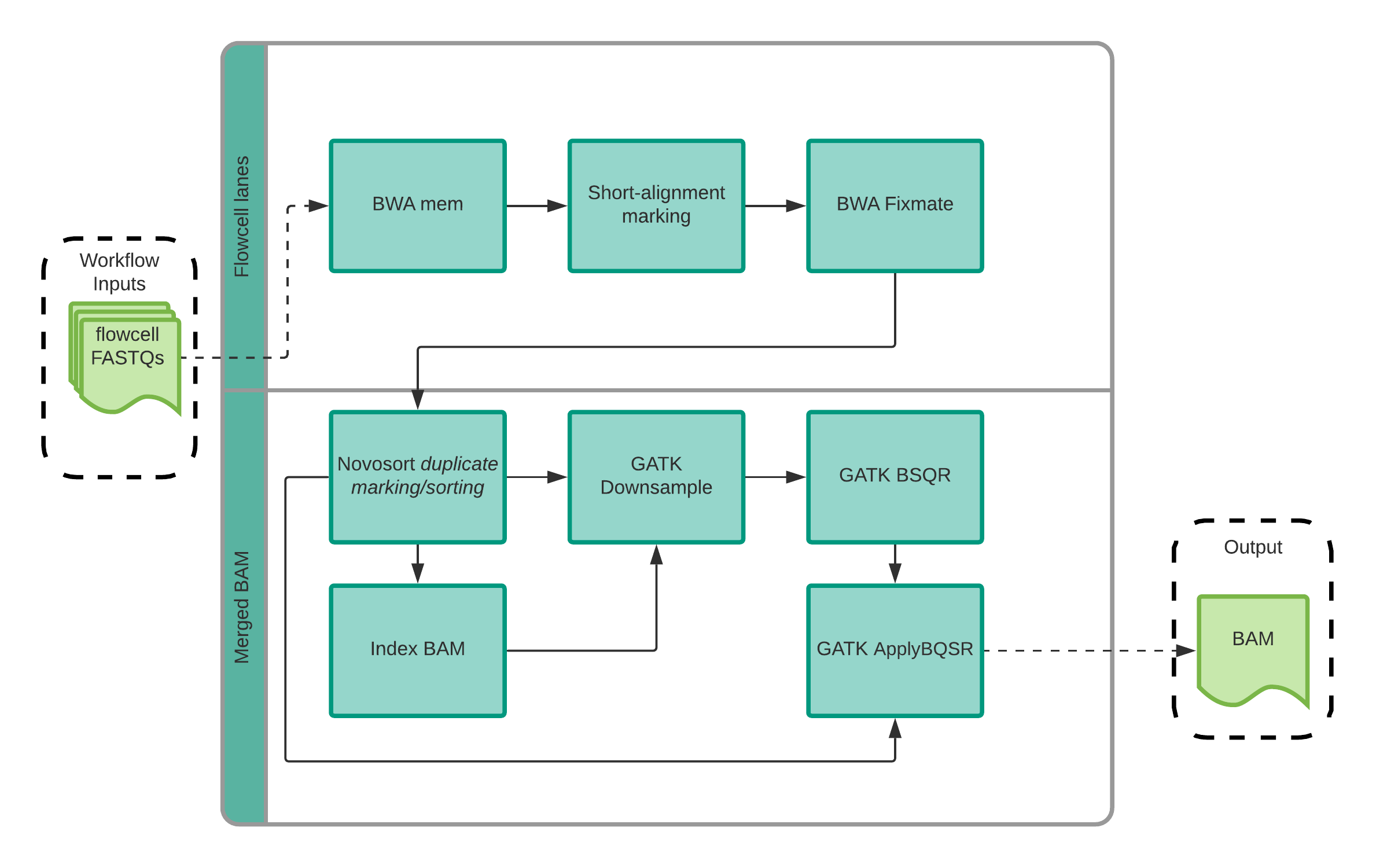

Supplement: Supplementary file 2 — Supplementary File [file 41598_2019_55636_MOESM2_ESM.zip › pipeline_specs/figs/WGS_preprocess.png]
